# From “Passive Supplementation” to “Active Repair”: Melatonin Reshapes the Treatment Paradigm for Late‐Onset Hypogonadism by Targeting Leydig Cell Senescence

**DOI:** 10.1111/acel.70492

**Published:** 2026-05-04

**Authors:** Hui Wu, Gang Ning, Mintao Jian, Ajian Peng, Haoyu Wang, Bonan Li, Xing Zhou

**Affiliations:** ^1^ Department of Andrology The First Hospital of Hunan University of Chinese Medicine Changsha China; ^2^ Department of Andrology Affiliated Changsha Hospital of Hunan Normal University Changsha China

**Keywords:** aging, late‐onset hypogonadism, Leydig cells, melatonin, testosterone supplementation therapy

## Abstract

In the context of global population aging, public health challenges due to aging are garnering significant attention. Late‐onset hypogonadism (LOH) is a common age‐related condition in men characterized by progressively decreasing serum testosterone levels with age, manifesting as sexual dysfunction, reduced physical vigor, and psychological or neurological abnormalities. Testosterone is synthesized primarily in testicular Leydig cells (LCs), and LC senescence during aging is key for suppressing testosterone production. This review systematically synthesizes the multidimensional molecular mechanisms underlying LC senescence. This process is driven primarily by oxidative stress (OS) and mitochondrial dysfunction and encompasses multiple interrelated layers, including epigenetic remodeling, the senescence‐associated secretory phenotype (SASP), stem Leydig cell (SLC) niche degradation, and disruption of intrinsic circadian rhythms. Exogenous testosterone supplementation therapy (TST) remains the mainstay of the clinical management of LOH; however, its long‐term use increasingly poses safety risks and is inherently limited to treating symptoms. Melatonin, a molecule with pleiotropic antiaging properties, including potent antioxidant effects and the ability to improve mitochondrial function, has the potential to synergistically antagonize the aforementioned multiple LC senescence pathways. By effectively mitigating LC aging and promoting endogenous testosterone synthesis, the use of melatonin could shift the treatment paradigm for LOH from “passive hormone supplementation” to “active cellular function repair”. This article recapitulates preclinical and preliminary clinical evidence on the efficacy of melatonin in treating testicular or LC dysfunction, validating its therapeutic promise, and proactively identifies critical directions for future translational research, providing valuable insights for the development of novel, etiology‐oriented therapeutic strategies for LOH.

## Graphical abstract

Melatonin restores testosterone production in aging Leydig cells by targeting key pathological drivers, particularly oxidative stress and mitochondrial dysfunction. This approach offers a fundamental strategy to reshape the Late‐onset hypogonadism treatment paradigm.

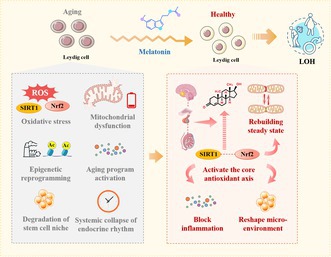

**Abbreviations**
17β‐HSD: 17β‐hydroxysteroid dehydrogenase
8‐OHdG: 8‐hydroxy‐2′‐deoxyguanosine
AhR: aryl hydrocarbon receptor
ALKBH5: AlkB homolog 5
BMAL1: brain and muscle ARNT‐like 1
BMI: body mass index
cAMP: cyclic adenosine monophosphate
CARM1: coactivator‐associated arginine methyltransferase 1
CAT: catalase
CCL2: C‐C motif chemokine ligand 2
cGMP: cyclic guanosine monophosphate
CYP11A1: cytochrome P450 family 11 subfamily A member 1
CYP17A1: cytochrome P450 family 17 subfamily A member 1
DEHP: di‐(2‐ethylhexyl) phthalate
DRP1: dynamin‐related protein 1
ECM: extracellular matrix
ED: erectile dysfunction
ERS: endoplasmic reticulum stress
FSH: follicle‐stimulating hormone
FTO: fat mass and obesity‐associated protein
GDF15: growth differentiation factor 15
GSH‐Px: glutathione peroxidase
hiPSCs: human induced pluripotent stem cells
HO‐1: heme oxygenase‐1
HPG: hypothalamic–pituitary–gonadal
HSD17B3: hydroxysteroid (17‐beta) dehydrogenase 3
HSD3B2: hydroxy‐delta‐5‐steroid dehydrogenase, 3 beta‐ and steroid delta‐isomerase 2
IL‐1β: interleukin‐1 beta
IL6: interleukin‐6
IL‐8: interleukin‐8
LCs: Leydig cells
LH: luteinizing hormone
LOH: late‐onset hypogonadism
m^6^A: N^6^‐methyladenosine
MDA: malondialdehyde
MEL1A: melatonin receptor 1A
MEL1B: melatonin receptor 1B
MERCs: mitochondria–endoplasmic reticulum contacts
METTL14: methyltransferase‐like 14
METTL3: methyltransferase‐like 3
Mff: mitochondrial fission factor
Mfn1: mitofusin 1
Mfn2: mitofusin 2
MMP13: matrix metalloproteinase‐13
MMP2: matrix metalloproteinase‐2
MMP3: matrix metalloproteinase‐3
MMP7: matrix metalloproteinase‐7
MMP8: matrix metalloproteinase‐8
MMP9: matrix metalloproteinase‐9
MMPs: matrix metalloproteinases
MT1: melatonin receptor 1
MT1‐MMP: membrane type 1 matrix metalloproteinase
MT2: melatonin receptor 2
MTNR1A: melatonin receptor 1A
mtROS: mitochondrial ROS
NQO1: NAD(P)H:quinone oxidoreductase 1
NRF1: nuclear respiratory factor 1
Nrf2: nuclear factor erythroid 2‐related factor 2
OIS: oncogene‐induced senescence
OPA1: optic atrophy 1
OPN: osteopontin
OS: oxidative stress
p38 MAPK: p38 mitogen‐activated protein kinase
PAI1: plasminogen activator inhibitor‐1
PARP‐1: poly(ADP‐ribose) polymerase −1
PER1: Period 1
PER2: Period 2
PINK1: PTEN‐induced putative kinase 1
PKA: protein kinase A
ROS: reactive oxygen species
SASP: senescence‐associated secretory phenotype
SA‐β‐Gal: senescence‐associated beta‐galactosidase
SCARB1: scavenger receptor class B member 1
SF‐1: steroidogenic factor 1
SLCs: stem Leydig cells
SOD: superoxide dismutase
StAR: steroidogenic acute regulatory protein
TAC: total antioxidant capacity
TERRA: telomeric repeat‐containing RNA
Tfam: mitochondrial transcription factor A
TGF‐β: transforming growth factor‐beta
TNFR1: tumor necrosis factor receptor 1
TNF‐α: tumor necrosis factor‐alpha
TSPO: translocator protein
TST: testosterone supplementation therapy
YTHDF3: YTH domain‐containing family protein 3

## 1 Introduction

The global trend of population aging is exacerbating the burden of age‐related diseases worldwide and has sparked widespread concern regarding aging‐related health issues. Late‐onset hypogonadism (LOH), an underrecognized public health problem, significantly affects the quality of life of middle‐aged and elderly men. In China, based on the diagnostic criterion of “a free testosterone concentration < 210 pmol/L accompanied by ≥ 3 sexual symptoms,” the prevalence of LOH among men aged 40–79 years is 7.8% (Liu et al. 2021). With the increasing aging population, this prevalence is expected to increase further. The core biochemical characteristic of LOH is the age‐related decrease in serum testosterone levels, which manifests as a clinical syndrome characterized by sexual dysfunction, diminished physical capacity, vasomotor disturbances, and neuropsychiatric abnormalities (Salonia et al. 2025; Snyder 2022). This condition is pathophysiologically rooted in the functional senescence of Leydig cells (LCs)—the primary functional units responsible for testosterone biosynthesis within the testicular compartment (Liu, Guo, et al. 2025). Testosterone secreted by LCs constitutes the fundamental hormonal milieu for maintaining secondary sexual characteristics, sexual function, musculoskeletal mass, and spermatogenesis in males (Galansky et al. 2022; Zhao, Li, et al. 2022).

During aging, LCs undergo a series of structural and functional degenerative changes, including reduced cell numbers, a diminished cell volume, and a significant decrease in the testosterone synthesis capacity (Chung et al. 2021; Mularoni et al. 2020; Neves et al. 2017; Xia et al. 2024). Underlying these phenotypic alterations is a complex molecular network: the accumulation of oxidative stress (OS) leads to lipid, protein, and DNA damage; mitochondrial dysfunction impairs the energy supply for steroidogenesis; and the downregulation of key steroidogenic enzymes (e.g., steroidogenic acute regulatory protein [StAR] and cytochrome P450 family 11 subfamily A member 1 [CYP11A1]) directly restricts testosterone biosynthesis. Collectively, these factors trigger and exacerbate LC apoptosis and cellular senescence (Kwon et al. 2025; Liu, Guo, et al. 2025; Luo et al. 2023). Importantly, senescent LCs may secrete the senescence‐associated secretory phenotype (SASP), which can amplify the aging process through paracrine effects that exacerbate testicular microenvironmental inflammation (Li, Zhang, et al. 2024). Therefore, LC senescence represents the fundamental cytopathological basis for the decrease in testosterone levels in individuals with LOH. Systematically elucidating its underlying molecular mechanisms and developing strategies to target, reverse, or delay this process have established a novel therapeutic paradigm for LOH.

Exogenous testosterone supplementation therapy (TST) remains a cornerstone in the clinical management of LOH and is effective at ameliorating key symptoms, including sexual dysfunction, reduced muscle mass, decreased bone density, and impaired quality of life (Corona et al. 2022; Cruickshank et al. 2024; Isidori et al. 2022; Kruse et al. 2020; Pencina et al. 2024; Rasmussen et al. 2024). Nevertheless, the clinical application of TST continues to face significant challenges. First, high‐quality randomized controlled trials have confirmed that short‐term TST in LOH patients does not significantly increase the incidence of adverse events in the prostate after individuals with a high prostate cancer risk are excluded (Bhasin et al. 2023). Furthermore, for patients with a low risk of prostate cancer who also present with low testosterone levels, initiating TST can effectively increase serum testosterone levels without causing statistically significant fluctuations in prostate‐specific antigen levels (Applewhite et al. 2025). These findings are attributable to stringent pretreatment screening and meticulous monitoring during therapy. Therefore, a comprehensive evaluation of cardiovascular safety, individualized prostate risk, and other potential health impacts is essential before initiating TST in clinical practice (Bhasin and Thompson 2024; Bhasin et al. 2023; Lincoff et al. 2023). Second, TST exerts negative feedback inhibition on the hypothalamic–pituitary–gonadal (HPG) axis, thereby suppressing spermatogenesis. This mechanism undoubtedly increases the treatment risk for men with fertility aspirations (Naelitz et al. 2025). Third, the absence of universally standardized diagnostic criteria for LOH introduces substantial heterogeneity into clinical decision‐making, often leading to either undertreatment of symptomatic individuals or overtreatment of patients with subclinical cases (Liu et al. 2021). Most critically, as an exogenous testosterone supplementation therapy, TST does not target the core pathology of LC senescence. It is unable to reverse the endogenous testosterone synthesis deficit and may even exacerbate the decline in intrinsic testicular function. Therefore, developing effective antiaging strategies is essential for the treatment of LOH (Tang et al. 2025). Precisely targeting the LC senescence process and implementing synergistic interventions involving multiple pathways to restore endogenous functional homeostasis represent emerging therapeutic strategies and key clinical focuses for treating LOH.

Melatonin, a rhythmic hormone primarily secreted by the pineal gland, has been increasingly recognized for its multifaceted antisenescence properties that extend far beyond its classical role in sleep regulation. Notably, it possesses robust capabilities to inhibit OS, improve mitochondrial function, and inhibit cellular senescence, with protective effects against specific cellular aging mechanisms that have been validated in tissues such as the retina and kidney (Cruciani et al. 2022; Hu et al. 2024; Zhang, Ding, et al. 2023). Notably, LCs express enzymes required for melatonin biosynthesis and melatonin receptors (Dong et al. 2020; Gao et al. 2019; Zhang et al. 2022), suggesting that they may be key target cells and peripheral sites for melatonin synthesis. Testosterone synthesis in LCs is highly susceptible to OS and mitochondrial dysfunction (Garza et al. 2022; Lu et al. 2022; Monageng et al. 2023), which are precisely the established core targets of melatonin. Therefore, we propose that melatonin likely exerts protective effects by modulating multiple molecular pathways centered on OS and mitochondrial function during LC senescence. By delaying the aging of LCs and fundamentally restoring their endogenous testosterone synthesis capacity, studies of melatonin provide novel insights and approaches for the precise clinical treatment of LOH.

## 2 Multidimensional Molecular Mechanisms of Leydig Cell Senescence

### 2.1 Oxidative Stress and Impaired Antioxidant Defense

OS, which is characterized by an imbalance between the pro‐oxidant and antioxidant systems in vivo, is recognized as a key mechanism mediating testicular aging (Han et al. 2023; Li et al. 2022). In aging LCs, OS disrupts cellular redox homeostasis and further impairs cell survival and testosterone synthesis through multiple synergistic pathways. As a central transcription factor in the antioxidant defense system, nuclear factor erythroid 2‐related factor 2 (Nrf2) ensures the effective clearance of excess reactive oxygen species (ROS) by upregulating the expression of various downstream antioxidant enzymes (Franci et al. 2024). Research has indicated that the downregulation of the deacetylase Sirt1 induces cellular senescence by increasing intracellular ROS generation and activating the p53 pathway (Liang et al. 2024). Sirt1 activation can partially mitigate the increased mitochondrial ROS levels by enhancing mitophagy (Zhang et al. 2021). Under physiological or various pathological conditions, the core antioxidant function mediated by Sirt1–Nrf2 signaling in LCs tends to be downregulated, leading to excessive ROS accumulation and subsequent cellular senescence (Chung et al. 2021; Huang et al. 2018; Liu, Guo, et al. 2025; Xia et al. 2024; Yu et al. 2025; Zheng et al. 2024).

DNA damage and mitochondrial dysfunction are often direct consequences of ROS accumulation (Prasad et al. 2025; Wang et al. 2021). In LCs, a deficiency in nuclear receptor subfamily 2 group C member 2 expression exacerbates DNA damage and is accompanied by multiple cellular senescence phenotypes, including increased senescence‐associated beta‐galactosidase (SA‐β‐Gal) activity (Dalian et al. 2026). These findings suggest that ROS accumulation resulting from Sirt1–Nrf2 inhibition ultimately drives LC senescence through the induction of DNA damage.

Physiological mitophagy normally helps alleviate nuclear DNA damage caused by mitochondrial ROS accumulation, thereby delaying cellular senescence (Franci et al. 2022). However, excessive ROS accumulation in cells can readily induce autophagic dysfunction, which in turn promotes and exacerbates cellular senescence (Qi, Yang, Xue, et al. 2024). Research has indicated that acrylamide can induce excessive autophagy in LCs by triggering OS, thereby leading to impaired cellular function (Meng et al. 2025). These findings suggest that autophagy dysfunction mediated by imbalanced OS may represent another key mechanism driving LC senescence. Moreover, OS impairs mitochondrial fusion in LCs by suppressing the expression of key fusion proteins—mitofusin 1 (Mfn1), mitofusin 2 (Mfn2), and optic atrophy 1 (Opa1)—thereby promoting apoptosis in LCs (Wang et al. 2023).

In addition, OS in LCs suppresses testosterone synthesis through multiple mechanisms. It not only directly downregulates the expression of StAR by inhibiting the cyclic adenosine monophosphate (cAMP)/protein kinase A (PKA)/steroidogenic factor 1 (SF‐1) signaling pathway (Yang, Liu, et al. 2025) but also contributes to the induction of apoptosis in LCs through various pathways (Grillo et al. 2024; Huang et al. 2024; Zhang, Ma, et al. 2023). These processes collectively lead to a loss of function during LC senescence. In summary, OS plays a central initiating and driving role in the aging of LCs. It not only directly causes nuclear senescence by inducing DNA damage but also drives the subsequent cascade of aging through synergistic mechanisms involving the disruption of mitochondrial homeostasis, interference with the autophagic balance, inhibition of testosterone synthesis, and induction of apoptosis.

### 2.2 Mitochondrial Dysfunction

Mitochondria, which serve as the central organelles for energy production and testosterone synthesis in LCs, play a pivotal role in cellular function. Their dysfunction impairs both energy metabolism and the steroidogenic capacity in LCs, representing a key mechanism underlying LC senescence (Li, Dong, et al. 2024).

During aging, mitochondrial function in LCs is comprehensively impaired, manifested as attenuated mitochondrial biogenesis (downregulation of mitochondrial transcription factor A (Tfam)–nuclear respiratory factor 1 (NRF1) signaling), suppressed mitophagy (downregulation of PTEN‐induced putative kinase 1 (PINK1)), and impaired mitochondrial fusion dynamics (downregulation of Mfn1 and Opa1) (Sokanovic et al. 2021).

Deficient expression of translocator protein (TSPO), a key cholesterol transport protein, not only limits the rate‐limiting step of steroidogenesis but also disrupts mitochondrial membrane dynamics, thereby further compromising mitochondrial functional integrity (Chung et al. 2013; Garza et al. 2022). Notably, enhancing mitochondrial fusion has been shown to effectively reverse mitochondrial morphological abnormalities and restore testosterone synthesis, highlighting the pivotal role of fusion dynamics in preserving the young functional state of LCs (Garza et al. 2022; Lv et al. 2024).

As mitochondria are the primary site of ROS generation within cells, the excessive accumulation of mitochondrial ROS (mtROS) often leads to irreversible senescence in LCs (Liu, Guo, et al. 2025). This accumulation is associated with dysregulation of the STAT5B–mitoSTAT3 signaling axis. This axis not only impairs mitochondrial respiratory chain function, thereby increasing ROS levels, but also induces cell cycle arrest by upregulating P21 expression, representing a key molecular event driving cellular senescence (Cao et al. 2024). Furthermore, excessive intracellular ROS accumulation induces the nuclear‐to‐cytoplasmic translocation of coactivator‐associated arginine methyltransferase 1 (CARM1). This process, in turn, leads to mitochondrial dysfunction through the methylation of dynamin‐related protein 1 (DRP1) and its enhanced interaction with mitochondrial fission factor (Mff), resulting in further ROS production (Cho and Kim 2024). This establishes a deleterious “ROS–mitochondrial damage–ROS” feedback loop that accelerates cellular senescence. A targeted reduction in mitochondrial ROS generation can effectively protect cellular DNA from damage while suppressing excessive autophagy and apoptosis (Park et al. 2022), representing a viable strategy to ameliorate nuclear senescence.

The evidence described above indicates that mitochondrial dysfunction is both a critical pathological target induced by OS and an important pathological source that perpetuates its exacerbation. The two mutually reinforce each other, forming a vicious self‐amplifying cycle that plays a key role in the age‐related decrease in testosterone synthesis. However, the specific regulatory mechanisms involved require further in‐depth investigation.

### 2.3 Epigenetic Remodeling

In addition to direct pathological damage, OS is capable of modulating LCs via epigenetic remodeling. N6‐methyladenosine (m6A), which is recognized as the most prevalent internal modification in eukaryotic mRNA, represents a pivotal component of the posttranscriptional regulation of gene expression (Jiang et al. 2021). In recent years, epigenetic mechanisms such as the m^6^A modification have been identified as critical regulators of testosterone synthesis in LCs (Xu et al. 2025). The dynamic equilibrium of m^6^A is coordinately maintained by methyltransferases (e.g., methyltransferase‐like 3 (METTL3) and methyltransferase‐like 14 (METTL14)) and demethylases (e.g., Fat mass and obesity‐associated protein (FTO) and AlkB homolog 5 (ALKBH5)). This modification exerts precise control over cellular homeostasis through its recognition by YTHDF family reader proteins.

Functional inactivation of the FTO–m^6^A axis serves as a critical hub in LC injury. The inhibition or knockdown of FTO results in elevated global m^6^A levels, which in turn leads to a marked decrease in testosterone secretion and an increase in the apoptosis of LCs (Wang et al. 2025; Zhao et al. 2021). Multiple environmental endocrine‐disrupting chemicals exacerbate LC injury by interfering with the FTO–m^6^A axis. Bisphenol F transcriptionally suppresses FTO expression via the aryl hydrocarbon receptor (AhR), leading to increased levels of the m^6^A modification on the Nrf2 mRNA. This modified transcript is subsequently recognized and degraded by YTHDF2, thereby compromising the antioxidant defense capacity of LCs, intensifying OS, and promoting apoptosis (Zhou et al. 2023). Similarly, triclosan downregulates FTO while upregulating YTHDF1, which increases the m^6^A‐dependent translation of the key autophagy gene *Becn1*, thereby inducing aberrant autophagy and ultimately inhibiting testosterone synthesis (Sang et al. 2024).

m^6^A RNA methylation bidirectionally regulates autophagy and OS pathways, thereby influencing LC function. Autophagy serves as a critical downstream target of the m^6^A modification and is differentially modulated under various physiological or pathological conditions. For instance, bisphenol A alters the expression of METTL3 and ALKBH5, increases global m^6^A levels in LCs, and specifically suppresses the expression of the key autophagy gene *Map1lc3b*, thereby impairing autophagic flux, accelerating cellular senescence, and reducing testosterone synthesis (Chen, Chen, et al. 2025). Conversely, under physiological conditions or upon gonadotropin stimulation, decreased METTL14 levels and increased ALKBH5 levels in LCs lead to reduced m^6^A methylation levels, which in turn activate protective autophagy via the AMPK pathway to support testosterone production (Chen et al. 2021).

Notably, the m^6^A modification in LCs is closely linked to OS. Increased m^6^A levels during LC injury are associated with various pathophysiological disturbances, including ROS accumulation (Zhao et al. 2021). FTO can upregulate Nrf2 expression by reducing its m^6^A modification, thereby counteracting bisphenol F‐induced oxidative damage and apoptosis in LCs (Zhou et al. 2023). More directly, OS mediates the upregulation of m^6^A modifications on key cholesterol transport genes, scavenger receptor class B member 1 (SCARB1) and StAR, upon bisphenol H exposure. These modifications are recognized by YTHDF3, leading to suppressed gene expression and the subsequent blockade of the initial steps of testosterone synthesis (Wang, Lu, et al. 2024).

Furthermore, Sirt1, a key factor involved in maintaining redox homeostasis in LCs, is itself regulated by the m^6^A modification (Wang et al. 2022). The upregulation of METTL3 increases m^6^A levels on the Sirt1 mRNA, which promotes cellular senescence and leads to a marked decrease in testosterone secretion (Zheng et al. 2024). Collectively, these findings demonstrate that m^6^A RNA methylation functions as a pivotal epitranscriptional hub in LCs by modulating core pathways such as autophagy and OS, thereby driving both cellular senescence and dysfunctional testosterone synthesis.

### 2.4 Senescence‐Associated Secretory Phenotype

Senescent cells secrete a spectrum of inflammatory factors, growth factors, and proteases, collectively known as the SASP (Wang, Han, et al. 2024). Under both physiological and pathological conditions, aging induces increased expression of p16 and p21 in LCs (Liu, Guo, et al. 2025; Luo et al. 2023; Zheng et al. 2024). The sustained activation of the p16 and p21 signaling pathways not only serves as a key indicator of cell cycle arrest but also functions as an upstream regulatory hub for SASP initiation. Specifically, p21 overexpression promotes the upregulation of multiple SASP components, including growth differentiation factor 15 (GDF15), matrix metalloproteinase‐2 (MMP2), matrix metalloproteinase‐3 (MMP3), osteopontin (OPN), plasminogen activator inhibitor‐1 (PAI1), tumor necrosis factor receptor 1 (TNFR1), tumor necrosis factor‐alpha (TNF‐α), interleukin‐6 (IL6), and C‐C motif chemokine ligand 2 (CCL2) (Englund et al. 2023). The upregulation of these proteins is likely to sustain a proinflammatory microenvironment around LCs, which may in turn exacerbate the cellular senescence process.

OS serves as a crucial driver in activating the SASP program. In gingival fibroblasts, ROS accumulation‐induced OS triggers cellular senescence and partially mediates the upregulation of SASP factors, including IL6, interleukin‐1β (IL‐1β), transforming growth factor‐beta (TGF‐β), and interleukin‐8 (IL‐8), during senescence (Guo et al. 2024). In endothelial cells, the mitochondrial antioxidant mitoquinol targets and reduces mtROS levels, which not only reverses the upregulation of the senescence markers p16 and p21 and the increase in SA‐β‐gal activity induced by doxorubicin but also suppresses the expression of SASP factors such as IL‐8, IL‐6, and MMP‐3, thereby effectively alleviating cellular senescence (Hossein et al. 2025).

In senescent LCs, the expression of p21 is regulated by p38 mitogen‐activated protein kinase (p38 MAPK). The genetic ablation of p38 has been shown to restore the testosterone synthesis capacity and reduce both the SA‐β‐Gal staining intensity and p21 protein levels (Luo et al. 2023). As a key stress‐responsive kinase, p38 MAPK activation is often triggered by OS (Hou et al. 2022; Yuanyuan et al. 2026). These findings suggest that in senescent LCs, OS induced by mtROS accumulation may upregulate p21 expression through p38 MAPK activation, representing a potential key regulatory mechanism. Additionally, IGFBP7, a core component of the SASP, is highly expressed in senescent LCs and may play an important role in mediating the senescence of neighboring healthy cells (He et al. 2024; Siraj et al. 2024).

Notably, a coculture model involving GC‐1 spg cells and senescent LCs revealed a significant increase in the amount of the IL‐6 protein secreted by senescent LCs into the coculture medium (Li, Zhang, et al. 2024). This observation confirms the active paracrine function of senescent LCs and suggests that the release of SASP factors such as IL‐6 may alter the microenvironment and function of neighboring germ cells. Collectively, these findings demonstrate that the OS‐driven SASP not only exacerbates LC senescence and impairs testosterone synthesis through autocrine signaling but also may disrupt spermatogenesis and testicular homeostasis via paracrine signaling. This integrated mechanism provides a plausible explanation for the clinical co‐occurrence of testosterone deficiency and reduced fertility commonly observed in patients with LOH.

### 2.5 Degradation of the Stem Leydig Cell Niche

SLCs serve as crucial progenitor cells in maintaining testicular endocrine homeostasis and can replenish and renew functional LCs through proliferation and differentiation. Studies have shown that SLC transplantation can not only successfully differentiate into mature LCs with testosterone‐synthesizing capacity but also effectively restore testosterone production (Chi et al. 2024; Zhang et al. 2017). However, aging significantly impairs SLC proliferation and differentiation (Shao et al. 2023).

During aging, the progressive increase in extracellular matrix (ECM) stiffness activates the mechanosensitive ion channel Piezo1, triggering sustained calcium influx. This cascade leads to mitochondrial dysfunction and excessive ROS generation. The elevated ROS levels subsequently promote the degradation of the transcription factor Gli1 via the ubiquitin–proteasome pathway, ultimately inhibiting the proliferation and differentiation of SLCs (Huang et al. 2025). These findings reveal the intermediate regulatory function of OS in the inhibition of the proliferation and differentiation of SLCs.

Matrix metalloproteinases (MMPs) serve as key effectors in the process of ECM remodeling (de Almeida et al. 2022; Wolosowicz et al. 2024). As a central mechanosensor, Piezo1 specifically upregulates the expression of various MMPs, including MMP2, MMP3, matrix metalloproteinase‐9 (MMP9), and membrane type 1 matrix metalloproteinase (MT1‐MMP), in a tissue‐specific manner. By precisely regulating ECM degradation and remodeling, Piezo1‐mediated MMP expression plays a critical regulatory role in maintaining aqueous humor outflow homeostasis, driving pathological remodeling in osteoarthritis, and promoting angiogenesis (Kang et al. 2019; Li, Hao, et al. 2024; Morozumi et al. 2022). In testicular tissue, OS upregulates the expression of MMP2 and MMP9, which cooperatively drive testicular cell apoptosis and tissue damage (Bajgiran et al. 2023). Although whether the Piezo1–MMP signaling axis directly regulates the degradation of the SLC niche remains unclear, the intracellular OS environment resulting from Piezo1 activation likely contributes to pathological ECM remodeling and the deterioration of the SLC niche microenvironment by modulating the expression and activity of MMPs.

Maintaining mitochondrial function is essential for stem cell vitality. In human mesenchymal stem cells, the Nestin protein is localized to mitochondria and interacts directly with Mic60, a key protein localized to mitochondrial cristae, thereby preserving mitochondrial structure and function and delaying cellular senescence (Hainan et al. 2025). Mitochondria–endoplasmic reticulum contacts (MERCs) play a pivotal role in regulating cellular senescence, and their dysfunction may lead to permanent proliferative arrest (Ziegler et al. 2021). In SLCs, the loss of Nestin disrupts the structure of MERCs, leading to dysregulated lipid transport and calcium homeostasis, thereby impairing the differentiation capacity of aged SLCs (Yao et al. 2022).

In summary, the current evidence suggests that aging may contribute to a decrease in the regenerative potential of SLCs through two distinct pathways: the Piezo1–MMP axis and Nestin protein‐mediated mechanisms. In this context, OS functions not only as a key downstream product of Piezo1 activation but also as a participant in ECM remodeling through MMP regulation while concurrently representing a core manifestation of mitochondrial dysfunction. These intertwined mechanisms collectively exacerbate the age‐related deterioration of the SLC niche.

### 2.6 Endocrine Circadian Rhythm Disruption

During LC aging, a systematic disruption of intrinsic endocrine rhythms occurs, and is primarily characterized by attenuated circadian oscillations in testosterone secretion (Baburski et al. 2016). The molecular foundation of this phenomenon is the widespread transcriptional downregulation of core clock genes—including brain and muscle ARNT‐like 1 (Bmal1), Period 1 (Per1), Period 2 (Per2), and Rev‐erbα—which weakens the central circadian drive. This impairment subsequently leads to the dysregulation of the downstream rhythmic output network, characterized by a substantially dampened oscillation amplitude of steroidogenic genes such as StAR, CYP11A1, and cytochrome P450 family 17 subfamily A member 1 (CYP17A1), as well as markedly weakened or even abolished transcriptional rhythms in pathways related to cholesterol uptake and mobilization (Baburski et al. 2016). Among these genes, the clock gene Bmal1 plays a crucial role in directly regulating the cyclic transcription of steroidogenic genes. Bmal1 knockout results in the comprehensive downregulation of the expression of steroidogenic genes, including StAR, CYP11A1, hydroxy‐delta‐5‐steroid dehydrogenase, 3 beta‐ and steroid delta‐isomerase 2 (HSD3B2), and hydroxysteroid (17‐beta) dehydrogenase 3 (HSD17B3), ultimately leading to reduced serum testosterone levels (Yang, Ma, et al. 2021).

During aging, the circadian clock system and OS engage in complex bidirectional regulation. On the one hand, OS serves as a key mechanism through which Bmal1 deficiency drives aging‐related pathologies, and interventions such as melatonin may exert their therapeutic effects by modulating Bmal1 expression (Fan et al. 2022). Specifically, the loss of Bmal1 leads to the dysregulation of the Nrf2/ARE pathway and downregulation of its downstream target gene *Prdx6* in senescent cells, thereby exacerbating ROS accumulation and cell death (Chhunchha et al. 2020). On the other hand, ROS accumulation induced by OS downregulates the expression of the key regulator EZH2, which impairs the transcriptional activation of the circadian gene *per1b* by the CLOCK–BMAL1 complex, ultimately resulting in circadian disruption (Zhang et al. 2025). These findings provide a novel mechanistic perspective on how OS contributes to the aging process.

While the role of the circadian clock in regulating OS and thereby affecting testosterone synthesis in LCs has been recognized (Ning et al. 2025), the underlying regulatory mechanisms and causal relationships between circadian transcriptional dysregulation and OS during LC aging remain to be systematically elucidated. Additionally, circadian disruption also induces mitochondrial dysfunction, suppressing the mitochondrial membrane potential and ATP production (Pavlovic et al. 2022), which further compromises the energy and substrate supply required for testosterone synthesis.

Notably, under conditions of circadian disruption, decreased circulating melatonin levels are associated with reduced serum testosterone concentrations (Travicic et al. 2025). These findings suggest that melatonin may act as a key signaling mediator involved in coordinating the intrinsic circadian rhythm with testosterone synthesis in LCs. In summary, the dysregulation of endocrine rhythms represents a core characteristic of LC aging, in which OS likely plays a dual role: serving as both a significant consequence of circadian disturbance and an upstream factor that further disrupts circadian homeostasis.

In summary, LC aging involves a self‐amplifying cascade driven by OS and exacerbated by mitochondrial dysfunction. This degenerative process is initiated by a dual mechanism: direct OS‐induced mitochondrial damage coupled with epigenetic modifications. On the one hand, OS impairs respiratory chain function, disrupts mitochondrial dynamics and quality control, and triggers excessive mitochondrial ROS production, establishing a vicious “OS–mitochondrial injury–further OS” feedback loop. On the other hand, OS‐mediated epigenetic alterations—such as the m6A modification—suppress the expression of steroidogenic genes (e.g., StAR) and compromise the Sirt1/Nrf2 antioxidant defense system. Mitochondria thus act as a central hub in LC aging, where their dysfunction not only reflects the consequences of OS but also perpetuates and amplifies cellular stress via excessive ROS generation and metabolic dysregulation, ultimately activating senescence pathways such as p38 MAPK/p21 signaling. Upregulated p21 not only induces cell cycle arrest but also promotes the SASP, which reinforces the senescent state of LCs through autocrine and paracrine signaling and contributes to the degradation of the SLC niche.

**FIGURE 1:**
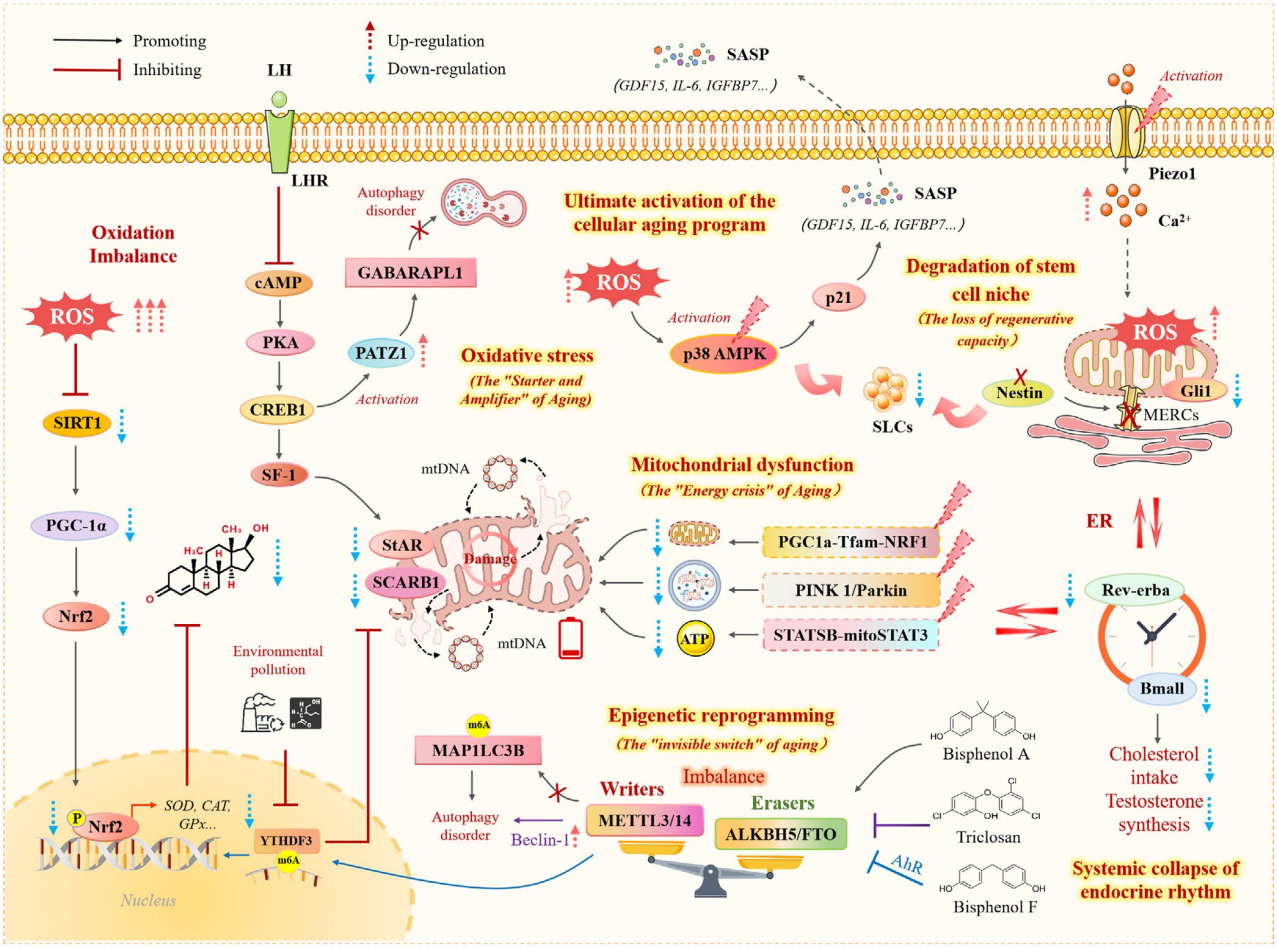
Multidimensional molecular mechanisms regulating LC senescence. LC aging is driven by a self‐amplifying positive feedback loop between OS and mitochondrial dysfunction. This core mechanism triggers multiple downstream events, including epigenetic reprogramming (e.g., dysregulated m^6^A modification), altered signaling pathways (suppression of Sirt1/Nrf2, activation of p38 MAPK/p21), induction of the SASP, and degradation of the SLCs niche. Collectively, these disturbances lead to attenuated oscillations of circadian clock genes and loss of rhythmic testosterone synthesis, marking the irreversible functional decline of LCs. This figure was created with Microsoft PowerPoint.

Collectively, these alterations lead to a systemic breakdown spanning the molecular, cellular, and microenvironmental levels, culminating in the global disruption of endocrine rhythms. Attenuated oscillations of core circadian genes desynchronize the steroidogenic program and abolish the circadian pattern of testosterone secretion, marking the transition to irreversible functional decline. Therefore, LC aging represents a prototypical positive‐feedback spiral in which OS and mitochondrial dysfunction serve as core initiators and amplifiers, interacting through multiple mechanisms, including epigenetic reprogramming, senescence‐associated secretory phenotype activation, niche deterioration, and circadian disruption, to drive the progressive failure of testosterone synthesis (Figure 1).

## 3 Multidimensional Mechanisms of Melatonin in Ameliorating Leydig Cell Senescence

### 3.1 Effect of Melatonin on Enhancing Antioxidant Defenses

During aging, plasma melatonin levels are significantly lower in aged mice than in young controls and are accompanied by increased OS, which is characterized by suppressed Nrf2 pathway activity and the excessive accumulation of ROS in cells (Wu et al. 2024). Melatonin exerts its cytoprotective effects primarily by mitigating OS through the activation of multiple signaling pathways in LCs. Studies have demonstrated that melatonin significantly reduces the intracellular levels of ROS, malondialdehyde (MDA), and 8‐hydroxy‐2′‐deoxyguanosine (8‐OHdG) while concurrently increasing the activities of key antioxidant enzymes such as superoxide dismutase (SOD) and glutathione peroxidase (GSH‐Px) (Chen, Tang, et al. 2025; Dong et al. 2020; Xu et al. 2019). A randomized controlled clinical trial confirmed that melatonin supplementation significantly increased the urinary excretion of 8‐OHdG in shift workers, suggesting increased clearance and repair of oxidative DNA damage in vivo (Umaimah et al. 2025). As a key biomarker of oxidative DNA damage, altered 8‐OHdG levels indicate that melatonin may help protect DNA from oxidative damage, thereby potentially contributing to the retardation of nuclear senescence in LCs.

In LCs, the antioxidant effect of melatonin relies on the activation of the AKT/Nrf2 signaling pathway: by promoting AKT phosphorylation, melatonin activates the transcription factor Nrf2 and upregulates its downstream target genes heme oxygenase‐1 (HO‐1) and NAD(P)H:quinone oxidoreductase 1 (NQO1), thereby systemically enhancing the cellular antioxidant defense capacity (Dong et al. 2020).

SIRT1 acts as a central hub in this process, serving as a key downstream effector through which melatonin ameliorates oxidative stress. It not only restores Nrf2 pathway activity to increase ROS clearance but also cooperatively promotes the functional recovery of key testosterone synthesis genes such as StAR and CYP11A1 (Chen, Tang, et al. 2025). Additionally, SIRT1 regulates Nrf2‐mediated antioxidant gene expression through PGC‐1α (Liu, Guan, et al. 2025). This protective effect also depends on the activation of melatonin membrane receptors; the inhibition of melatonin receptor 1 (MT1)/melatonin receptor 2 (MT2) not only blocks the activation of SIRT1/Nrf2 signaling but also exacerbates apoptosis (Zhang et al. 2022). SIRT1‐mediated antioxidant repair in LCs may thus create a favorable microenvironment for subsequent peripheral melatonin synthesis, although this hypothesis requires further investigation.

In addition to its direct antioxidant actions, melatonin indirectly suppresses various OS‐triggered cell death pathways. For example, melatonin can inhibit excessive autophagy in LCs induced by dibutyl phthalate exposure (Yang, Yang, et al. 2025) and alleviate ferroptosis in LCs by suppressing the OS–Sp2/VDAC2 axis (Yang et al. 2024). Collectively, these findings indicate that melatonin plays a pivotal role in repairing oxidative damage and improving LC function through the multitargeted regulation of cellular redox homeostasis.

### 3.2 Effect of Melatonin on Restoring Mitochondrial Function

Melatonin exerts multi‐dimensional protective effects on restoring mitochondrial function in LCs, primarily through the reversal and recovery of mitochondrial biogenesis, autophagy, dynamics, and quality control, which are impaired by OS. Studies indicate that this protective effect is closely associated with the activation of SIRT1‐related signaling pathways and the restoration of redox homeostasis. Specifically, melatonin activates the SIRT1/PGC‐1α axis to upregulate Nrf2 activity and increase ROS clearance (Liu, Guan, et al. 2025) while preserving mitochondrial quality and protecting steroidogenic function in LCs via the AMPK/SIRT1 pathway (Wang et al. 2022).

Furthermore, under specific pathological conditions, melatonin inhibits aberrant mitophagy. Through its antioxidant actions, melatonin specifically suppresses excessive Parkin‐mediated mitophagy, thereby alleviating glyphosate‐induced inhibition of testosterone synthesis (Ren et al. 2024).

In summary, melatonin systematically improves mitochondrial function in LCs across multiple dimensions—biogenesis, homeostasis of mitochondrial dynamics, and quality control. Throughout this process, its antioxidant effects, which are mediated by clearing ROS and mitigating oxidative damage, provide crucial synergistic support for the comprehensive restoration of mitochondrial function. Together, these mechanisms constitute a key pathway through which melatonin delays LC senescence.

### 3.3 Effects of Melatonin on Restoring the Stem Leydig Cell Niche

In the context of aging, a decrease in the regenerative capacity of SLCs represents a major contributor to testosterone deficiency. Melatonin can reconstruct the Nestin‐mediated structure of MERCs in aged SLCs. This pivotal effect directly restores the differentiation potential of SLCs, thereby providing a sustainable cellular source for increasing circulating testosterone levels (Yao et al. 2022). Although the regulatory effects of melatonin on MMPs in LCs remain incompletely elucidated, melatonin effectively suppresses the expression of matrix metalloproteinase‐7 (MMP7), MMP2, MMP9, MMP3, and matrix metalloproteinase‐13 (MMP13) in other pathological contexts (Nguyen et al. 2023; Yu et al. 2022; Zhao, Song, et al. 2022). Notably, a clinical trial involving patients with diabetic periodontitis showed that adjunct melatonin supplementation (6 mg daily for 30 days) significantly reduced matrix metalloproteinase‐8 (MMP8) levels in gingival crevicular fluid following nonsurgical periodontal therapy (Gul et al. 2024). Collectively, these findings not only suggest that melatonin may modulate MMPs at the molecular level to remodel the ECM and subsequently restore the regenerative and differentiation potential of SLCs but also clinically confirm its ability to directly regulate MMPs, providing translational insights for future therapeutic applications of melatonin.

In diabetic models, a melatonin intervention promotes the self‐renewal of spermatogonial stem cells (Du et al. 2018). These findings indicate that melatonin also supports the maintenance of spermatogonial stem cell homeostasis, thereby ensuring the orderly and stable progression of spermatogenesis. Moreover, testosterone synthesis in LCs depends on the involvement and regulation of Sertoli cells. Research has shown that in Sertoli cell–LC coculture systems, melatonin increases testosterone production by modulating Sertoli cell function and inhibiting local estrogen synthesis (Deng et al. 2018). Furthermore, the regulatory effect of melatonin on Sertoli cell function partially relies on the activation of the SIRT1/Nrf2 pathway (Qin et al. 2023). These findings indicate that melatonin plays a crucial signaling role in mediating functional crosstalk between Sertoli cells and LCs.

In summary, melatonin is likely to exert a synergistic regulatory effect on maintaining the overall homeostasis of the testicular niche. By concurrently supporting the function and stability of SLCs, spermatogonial stem cells, and Sertoli cells, melatonin provides protection and facilitates key testicular physiological processes, including testosterone synthesis and spermatogenesis.

### 3.4 Effect of Melatonin on Suppressing the Senescence‐Associated Secretory Phenotype

Melatonin clearly exerts anti‐inflammatory and SASP‐ameliorating effects to regulate LC senescence. Studies have shown that a melatonin intervention significantly suppresses the activation of the TLR4/NF‐κB signaling pathway in H₂O₂‐treated LCs and downregulates the expression of the downstream inflammatory cytokines IL‐6 and IL‐1β, effects that have been confirmed to depend on the activation of the SIRT1 pathway (Chen, Tang, et al. 2025). In an amyloid‐β‐induced senescence model of human neural stem cells, SIRT1 overexpression markedly attenuated the upregulation of p16 and p21 expression and reduced SA‐β‐gal activity (Li, Li, et al. 2024). These findings suggest that the effect of melatonin on modulating the SASP in LCs may be partially mediated by SIRT1.

While current research on the mechanisms underlying the effects of melatonin on LC senescence remains limited, its role in ameliorating the SASP has been further elucidated in broader models of aging. For instance, in oncogene‐induced senescence (OIS) models, melatonin significantly blocks the upregulation of senescence marker genes (p53, p21, and p16) and key SASP factors (CXCL1, IL6, IL8, and MMP3) by inhibiting the ROS–YTHDF2–MAPK–NF‐κB signaling axis, thereby effectively delaying senescence in human ovarian epithelial cells (Zhu, Ji, et al. 2022). Further mechanistic investigations revealed that melatonin dually suppresses the SASP through a poly(ADP‐ribose) polymerase‐1 (PARP‐1)‐mediated epigenetic pathway: it disrupts the telomeric repeat‐containing RNA (TERRA)–PARP‐1 interaction while simultaneously inhibiting histone H2BK120 acetylation by the macroH2A1.1–PARP‐1–CBP complex, consequently suppressing the transcriptional activation of SASP genes (Yu et al. 2017). These findings provide compelling evidence supporting the potential of melatonin as a multitarget antisenescence agent. The modulation of the “OS–epigenetics–inflammatory pathway” network represents a promising mechanism by which melatonin may suppress the SASP and delay LC aging.

### 3.5 Effect of Melatonin on Regulating Steroidogenic Gene Expression

Impaired steroidogenic pathway function represents a key mechanism underlying the decrease in testosterone synthesis during LC aging. Research has demonstrated that aging is associated with the significant downregulation of key steroidogenic genes, including STAR, CYP11A1, 3β‐HSD, CYP17A1, and 17β‐hydroxysteroid dehydrogenase (17β‐HSD), directly compromising the testosterone biosynthetic capacity (Li, Dong, et al. 2024; Yu et al. 2025). Melatonin has been shown to modulate the expression of steroidogenic genes in LCs and upregulate StAR, CYP17A1, and 17β‐HSD levels, thereby increasing testosterone synthesis (Liu, Guan, et al. 2025; Qi, Yang, Li, et al. 2024). Notably, this stimulatory effect depends on SIRT1 activity, as the pharmacological inhibition of SIRT1 completely abrogates the melatonin‐induced upregulation of these genes (Chen, Tang, et al. 2025), underscoring the integral role of the antioxidant function of melatonin in preserving steroidogenic competence.

Melatonin receptors serve as critical intermediaries in mediating the regulatory functions of melatonin. The melatonin receptor 1A (MTNR1A) is highly expressed in LCs, and its knockdown leads to reduced expression of key enzyme‐encoding genes such as StAR, CYP11A1, and CYP17A1, consequently significantly inhibiting testosterone production (Gao et al. 2019). Additionally, the nuclear receptor RORα is involved in melatonin‐mediated positive regulation of the steroidogenic pathway; its activation upregulates the expression of transcription factors, including StAR and SF1, thereby promoting testosterone production, whereas the blockade of RORα reverses melatonin‐associated effects (Yang, Guan, et al. 2021). These findings suggest that melatonin likely regulates steroidogenic gene expression through the coordinated actions of multiple receptors, thereby maintaining or restoring testosterone synthesis in LCs.

### 3.6 Regulatory Effect of Melatonin on the Hypothalamic–Pituitary–Gonadal Axis

Testosterone synthesis in LCs is regulated by the HPG axis (Cheng et al. 2024). Under physiological conditions, luteinizing hormone (LH) stimulates testosterone production in LCs, serving as a central mechanism for maintaining normal testosterone levels (Lei et al. 2025). Studies have shown that exogenous melatonin supplementation positively modulates the function of the HPG axis under various physiological and pathological conditions. Exogenous melatonin significantly increased the serum levels of LH and testosterone in prepubertal rat models, thereby promoting the normal onset of puberty (Venugopal 2019). In heat‐stressed goats, a single melatonin injection effectively increased local testicular levels of follicle‐stimulating hormone (FSH) and testosterone (Samir et al. 2023). In a rat hyperprolactinemia model, exogenous melatonin successfully reversed the low testosterone status and hypogonadism (de Araújo Silva et al. 2023). These observations suggest that melatonin may serve as an important regulator of the HPG axis, acting at the central level to modulate testosterone synthesis.

Notably, the regulatory effect of melatonin on the HPG axis is not simple unidirectional stimulation but rather a dynamic adjustment that depends on the physiological or pathological context. In a di‐(2‐ethylhexyl) phthalate (DEHP)‐induced testicular toxicity model, melatonin successfully increased serum testosterone levels without further increasing LH; instead, it suppressed the pathological increase in LH levels (Bahrami et al. 2018). These findings suggest that the effects of melatonin may extend beyond merely enhancing upstream LH signaling and may involve restoring the homeostasis of the HPG axis in response to stress or toxin exposure, thereby achieving the precise regulation of testosterone synthesis.

In summary, melatonin exerts precise regulatory effects on multiple key nodes involved in the molecular mechanisms underlying LC senescence, thereby effectively delaying the aging process. Its core actions involve simultaneous intervention in two central drivers of aging: OS and mitochondrial dysfunction. On the one hand, melatonin directly enhances cellular antioxidant defenses by activating the SIRT1–Nrf2 axis, thereby blocking the amplification cascade of OS (Chen, Tang, et al. 2025; Liu, Guan, et al. 2025); on the other hand, it comprehensively restores mitochondrial biogenesis, dynamics, and quality control, reestablishing the organellar basis for energy metabolism and steroidogenesis (Liu, Guan, et al. 2025).

Concurrently, melatonin significantly attenuates the inflammatory microenvironment mediated by the SASP by suppressing signaling pathways such as NF‐κB (Zhu, Ji, et al. 2022). It also reconstructs the stem cell niche and promotes the regenerative potential of SLCs, thereby maintaining the homeostasis of the LC pool at its source (Yao et al. 2022). Furthermore, as an endogenous circadian regulator, melatonin may synchronize the intrinsic circadian clock of LCs, restore the rhythmic transcription of steroidogenic genes, and dynamically modulate HPG axis function at the central level (Samir et al. 2023; Venugopal 2019), thereby promoting testosterone synthesis under physiological conditions and restoring homeostasis under pathological conditions.

**FIGURE 2:**
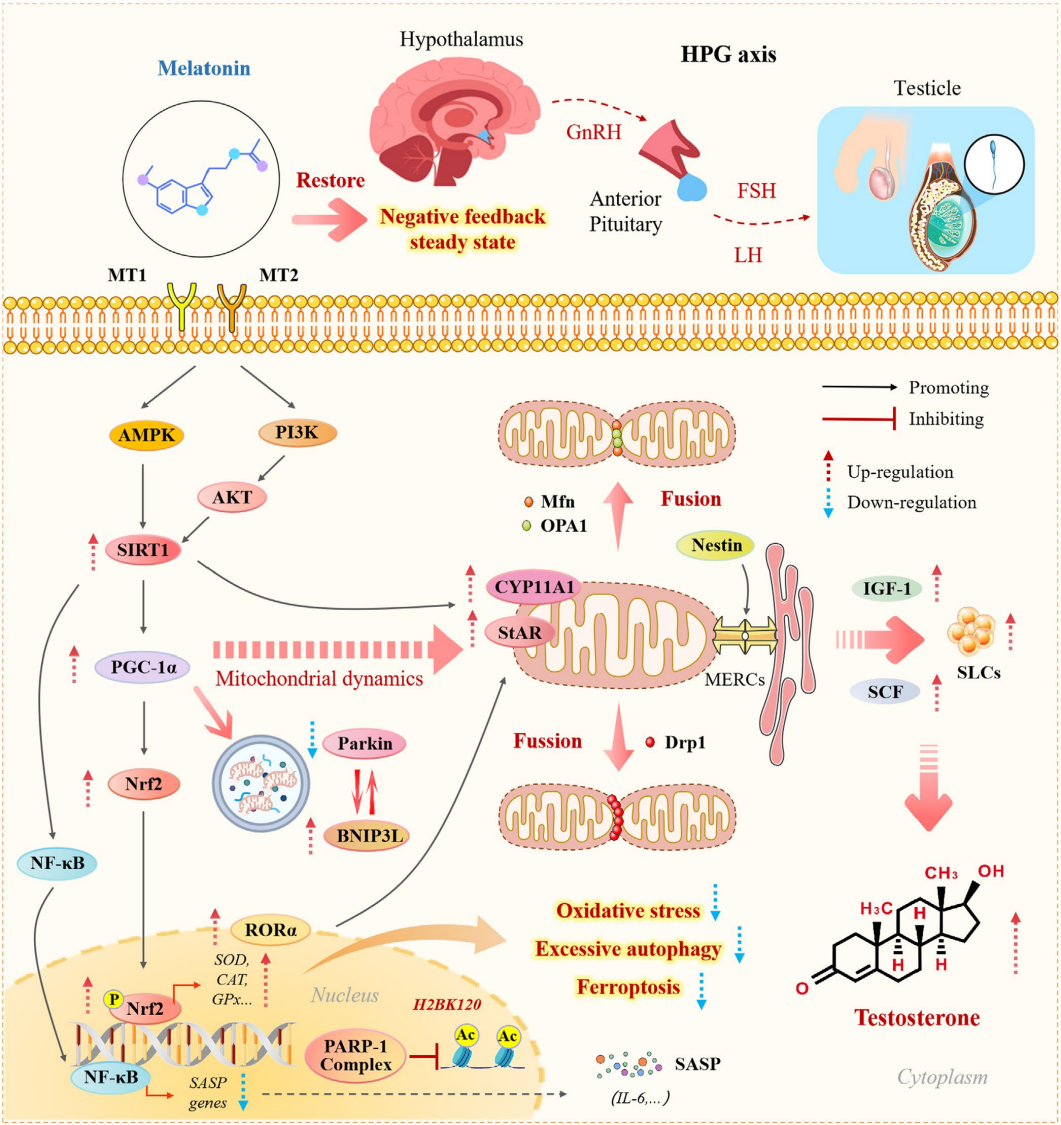
Mechanisms by which melatonin regulates LC senescence. On one hand, melatonin enhances antioxidant defense and suppresses the OS cascade by activating the SIRT1–Nrf2 axis; on the other hand, it restores mitochondrial biogenesis, dynamics, and quality control through the SIRT1–PGC‑1α pathway, thereby improving mitochondrial function. Concurrently, melatonin attenuates the inflammatory microenvironment mediated by the SASP by inhibiting NF‑κB signaling and promotes SLCs regeneration. Moreover, as an endogenous circadian regulator, melatonin synchronizes the intrinsic rhythm of LCs, restores the rhythmic transcription of steroidogenesis‐related genes, and facilitates physiological testosterone synthesis via modulation of the HPG axis. These multifaceted actions collectively constitute a synergistic regulatory network through which melatonin counteracts LC aging. This figure was created with Microsoft PowerPoint.

Collectively, melatonin not only interrupts the cascading process of LC senescence at the molecular and cellular levels through multidimensional actions but also integrates antioxidant, mitochondrial protective, anti‐inflammatory, pro‐regenerative, and neuroendocrine regulatory effects at the tissue and systemic levels, forming a well‐orchestrated network of synergistic mechanisms. These mechanistic insights provide a solid experimental foundation for the potential application of melatonin as an antiaging intervention in patients with LOH and underscore its considerable promise for clinical translation (Figure 2).

## 4 Translational Potential of Melatonin: From Animal Models to Clinical Applications

### 4.1 Preclinical Studies

Although intervention studies with melatonin in natural aging‐related LOH models remain at a preliminary exploratory stage, substantial evidence from studies using induced testicular or LC dysfunction models provides compelling and reproducible experimental data supporting the efficacy of melatonin (Dehdari Ebrahimi et al. 2023). Melatonin exerts its core protective effects primarily through its potent antioxidant capacity. In multiple in vivo and in vitro models, it systematically ameliorates OS and significantly inhibits apoptosis by activating the SIRT1–Nrf2 signaling axis (Dong et al. 2020; Liu, Guan, et al. 2025; Ma et al. 2021; Xu et al. 2019; Yang et al. 2023). Under distinct pathological conditions, melatonin exerts protective effects through actions on multiple pathways that ultimately promote testosterone synthesis. In type 2 diabetes models, its effects are closely associated with the alleviation of OS (Liu et al. 2022); in high‐fat diet‐induced testicular injury, melatonin primarily exerts protective effects by improving lipid metabolism (Saidi et al. 2022). Furthermore, in a complex model combining type 2 diabetes with sleep fragmentation, melatonin can reverse testicular lipid deposition and apoptosis (Zhang, Tang, et al. 2025).

In terms of environmental pollutant‐induced testicular injury, melatonin consistently produces reliable antagonistic effects. In bisphenol A and lead acetate exposure models, it mitigated oxidative damage and promoted the recovery of testosterone synthesis by inhibiting ERS and blocking the NF‐κB inflammatory pathway, respectively (Qi, Feng, Liu, et al. 2024; Qi, Yang, Li, et al. 2024; Zhao et al. 2023). In bisphenol S exposure models, melatonin achieved synergistic protective effects and increased testosterone synthesis through activation of the Nrf‐2/HO‐1 and SIRT‐1/FOXO‐1 antioxidant pathways while suppressing the NF‐κB/COX‐2 inflammatory pathway (Kumar et al. 2021; Sahu and Verma 2023). Moreover, the mechanisms underlying the effects of melatonin have been validated in depth at the epigenetic and signal transduction levels. It can mitigate pyroptosis induced by 1,2‐dichloroethane exposure by suppressing the piR‐mmu‐1019957–IRF7 axis (Zhang et al. 2024) and alleviate dysregulated autophagy induced by dibutyl phthalate exposure through the modulation of the Sp2/DAPK3 and PRKN/DAPK3 pathways (Yang, Yang, et al. 2025).

Notably, melatonin has shown remarkable translational potential in large animal models (such as goats), in which a single administration significantly improved testicular blood flow and increased testosterone levels (Samir et al. 2020; Shahat et al. 2022). Collectively, these findings indicate that through the coordinated regulation of multidimensional networks involving antioxidant, anti‐inflammatory, antiapoptotic, metabolic, and epigenetic mechanisms, melatonin exhibits robust protective efficacy and reliable clinical translation prospects across various preclinical pathological models of LOH.

In addition to its central protective role in LCs, the ability of melatonin to repair and improve the testicular microenvironment also merits significant attention. In Sertoli cells, melatonin acts through its membrane receptors MT1/MT2 to enhance cellular OS defenses by upregulating key antioxidant enzymes, including SOD1, catalase (CAT), and peroxiredoxin 1, while simultaneously stimulating the expression and activity of lactate dehydrogenase to significantly increase intracellular lactate production, thereby providing essential nutritional support for spermatogenic cells (Rossi et al. 2023). Additionally, studies have confirmed that melatonin ameliorates the disruption of mitochondrial flux mediated by the ROS–DRP1 axis, effectively reducing heat stress‐induced Sertoli cell apoptosis and maintaining the structural and functional integrity of Sertoli cells (Wen et al. 2025). Within the spermatogenic cell lineage, melatonin similarly exerted clear protective effects. In both spermatogonial stem cells and spermatocytes, melatonin significantly suppresses inflammation and ERS triggered by lipopolysaccharide or heat stress, thereby effectively blocking subsequent apoptotic pathways and maintaining spermatogenic homeostasis (Qin et al. 2021; Yang, Wei, et al. 2021). These findings collectively indicate that melatonin participates in maintaining testicular microenvironmental homeostasis by targeting multiple cell types.

Research involving human testicular tissue specimens has revealed that testicular aging involves alterations throughout the testicular microenvironment, including reduced Sertoli cell numbers and functional disturbances, decreased testosterone synthesis in LCs, peritubular cell hyperplasia or basement membrane thickening, weakened intercellular interactions, and the upregulation of inflammation‐related genes (Cui et al. 2025). These findings suggest that melatonin has substantial clinical potential for addressing disruptions in microenvironmental homeostasis during testicular aging.

While mechanistic evidence from various animal models suggests the unique potential of melatonin for clinical translation in LOH treatment, several critical challenges must be resolved before its clinical implementation. First, most evidence originates from pathology‐induced models in young animals, and its efficacy and safety in naturally aged populations has not been verified. Second, the dose–response relationship, optimal administration protocol (including timing and duration), and long‐term therapeutic effects of melatonin need standardization, particularly considering potential variations in the response of etiologically diverse populations of patients with LOH. Finally, the effect of exogenous supplementation on the regulation of endogenous melatonin secretion remains unclear. These aspects constitute crucial directions for future fundamental LOH research (Table 1).

**TABLE 1:** Preclinical studies on the role of melatonin in ameliorating interstitial cell senescence.

Summary of studies investigating melatonin using animal and experimental models	Optimal intervention concentration	Mechanism	Key findings	References
STZ‐treated rats and high glucose‐induced TM3 Leydig cells model	10 mg/kg/d	Activation of the AMPK/SIRT1 signaling axis	Upregulation of StAR, 3β‐HSD, and P450scc expression levels; improved mitochondrial function and autophagic activity; restoration of testosterone levels	(Wang et al. 2022)
H_2_O_2_‐induced rooster Leydig cell model	100 ng/mL	Activation of the AKT–Nrf2 signaling pathway	Amelioration of OS and mitochondrial dysfunction, suppression of apoptosis	(Dong et al. 2020)
TM3 Leydig cell model	10 ng/mL	Activation of the SIRT1 signaling pathway	Promotion of cell proliferation, suppression of apoptosis, amelioration of OS	(Xu et al. 2019)
4‐Methyl‐2,4‐bis(4‐hydroxyphenyl)pent‐1‐ene‐treated TM3 Leydig cell model	20 mg/kg (in vivo); 1 μM to 5 μM (in vitro)	Activation of the SIRT1/PGC‐1α signaling pathway	Restoration of mitochondrial function, amelioration of OS, suppression of apoptosis, promotion of testosterone synthesis	(Liu, Guan, et al. 2025)
Diquat‐induced male mouse model	10 mg/kg b.w./day	—	Attenuation of OS, suppression of apoptosis, and maintenance of blood–testis barrier integrity	(Yang et al. 2023)
Energy restriction‐induced sheep Leydig cell model	10 ng/mL	Activation of the SIRT1/SOD2 pathway	Amelioration of OS, promotion of testosterone secretion	(Ma et al. 2021)
High fat diet (FD)‐induced obese rat model	4 mg/kg	Activation of the PPAR‐γ signaling pathway	Improvements in lipid metabolism and OS, Increase in testosterone levels	(Saidi et al. 2022)
Male mice with type 2 diabetes mellitus combined with sleep fragmentation	10 mg/kg	Activation of FGFR1, blockade of the TAB1–TAK1 interaction, inhibition of TAK1 phosphorylation	Reduced lipid deposition and apoptosis	(Zhang, Tang, et al. 2025)
Bisphenol A‐induced mouse model	Injected with 20 mg/kg	—	Upregulation of melatonin receptor expression in testicular cells, suppression of ER S‐induced apoptosis, increased testosterone levels	(Qi, Feng, Liu, et al. 2024)
Lead acetate‐induced male mouse model	Intraperitoneal injection of 5 mg/kg	Inhibition of the NF‐κB signaling pathway	Amelioration of OS; improvement in the inflammatory response	(Zhao et al. 2023)
Bisphenol S‐exposed adult male golden hamster model	10 mg/kg BW/alternate days	Activation of the Nrf‐2/HO‐1 and SIRT‐1/FOXO‐1 antioxidant pathways, suppression of the NF‐κB/COX‐2 inflammatory pathway	Ameliorated OS and inflammation, increased serum testosterone levels	(Kumar et al. 2021)
1,2‐Dichloroethane‐treated mice and GC‐2 spd cell model	125 μM (in vitro)；20 mg/kg (in vivo)	Inhibition of the piR‐mmu‐1,019,957/IRF7 signaling axis	Alleviation of pyroptosis	(Zhang et al. 2024)
Dibutyl phthalate‐treated mice and mouse TM3 cell model	10 mg/kg	Suppression of OS with the concurrent blockade of Sp2‐mediated DAPK3 transcriptional upregulation and PRKN‐mediated impairment of DAPK3 degradation	Attenuation of autophagy in LCs	(Yang, Yang, et al. 2025)
Arylalkylamine N‐acetyltransferase (AANAT)‐overexpressing transgenic sheep and AANAT knockout mice	—	Modulation of the upregulation of key testosterone synthesis‐related genes, including StAR, SF1, and GATA‐4, mediated by the nuclear receptor RORα	Reduction in LC apoptosis, improvement in sperm quality, increase in testosterone levels	(Yang, Guan, et al. 2021)
Sexually mature male Shiba goats or mild heat stress‐induced yearling Dorset ram model	36 mg/goat	—	Improvement in testicular blood flow, increase in testosterone levels	(Samir et al. 2020; Shahat et al. 2022)

### 4.2 Clinical Investigations

Although extensive preclinical studies have revealed the potential of melatonin to protect testicular and LC function, clinical research targeting testicular or LC injury‐related disorders remains considerably limited. Nevertheless, the limited yet compelling clinical evidence provides substantial translational potential for the application of melatonin as a treatment for LOH. A pivotal retrospective clinical study demonstrated that infertile men with spermatogenic impairment who received 3 mg/day melatonin (for ≥ 3 months) for sleep disorders exhibited significantly favorable changes in testicular tissue, such as elevated melatonin concentrations, reduced total macrophage and interstitial macrophage counts, downregulated expression of key inflammatory factors (COX‐2, NLRP3, IL‐1β, and TNF‐α), thinning of the seminiferous tubule wall, and markedly decreased levels of the OS marker MDA (Riviere et al. 2020). These findings provide evidence that exogenous melatonin can cross the blood–testis barrier and exert core anti‐inflammatory and antioxidant effects locally within the testes. Concurrently, infertile men undergoing varicocelectomy who received 400 mg/day oral melatonin for 3 months presented significantly improved sperm parameters (sperm count, percentage of motile sperm, and proportion of sperm with a normal morphology), increased inhibin B levels, reduced sperm MDA levels, and an increased total antioxidant capacity compared with those in the placebo‐controlled group (Lu et al. 2018). In patients with benign prostatic hyperplasia, 1 month of combined therapy with 3 mg of oral melatonin and tamsulosin significantly improved nocturnal urination and urinary frequency symptoms compared with those in the control group (Fotovat et al. 2022).

These systemic benefits of melatonin are further supported by clinical findings from other medical fields. A systematic review and meta‐analysis showed that melatonin supplementation significantly improved OS parameters in individuals with a suboptimal health status compared with those in control groups (Morvaridzadeh et al. 2020), reinforcing the recognition of melatonin as an effective endogenous antioxidant. Multiple randomized controlled trials have consistently shown that melatonin supplementation significantly improves systemic OS status of patients with cardiovascular and neurological disorders, such as by increasing the total antioxidant capacity (TAC) and reducing MDA levels, and attenuates the expression of inflammatory markers, including TNF‐α (Mohammadi et al. 2025). Furthermore, melatonin administration has been shown to improve sleep quality, quality of life, muscle strength, and even mood and cognitive function (Jallouli et al. 2025; Sugumaran et al. 2024).

Hypogonadism, one of the most prominent symptoms of LOH, often causes significant distress to patients. Compared with healthy individuals, patients with erectile dysfunction (ED) exhibit significantly lower serum melatonin levels (Bozkurt et al. 2018). The therapeutic potential of exogenous melatonin for ED has been experimentally validated in various disease models, including chronic ischemia, diabetes, and hyperhomocysteinemia. In these models, exogenous melatonin restored erectile function by repairing structural and functional damage to penile tissues, ameliorating OS‐induced damage, reducing apoptosis, and suppressing inflammatory responses (Niu et al. 2024; Sawada et al. 2016; Tang et al. 2019).

These limited yet methodologically diverse clinical investigations provide a preliminary clinical rationale for considering melatonin therapy for patients with LOH, particularly those presenting with core pathological features of OS and chronic inflammation, along with characteristic symptoms such as sleep disturbances, mood alterations, decreased muscle strength, nocturia or urinary frequency, and ED.

## 5 Limitations in Melatonin Application

### 5.1 Species‐Specific Variations

The regulation of testosterone synthesis by melatonin is markedly species‐ and context dependent, as it does not have a simple stimulatory or inhibitory effect but rather involves a highly complex regulatory network. In rooster models (typical photoperiod‐dependent breeding animals), multiple studies have consistently described the inhibitory effect of melatonin on testosterone synthesis, involving multiple molecular pathways. Mechanistically, melatonin exerts a dual inhibitory effect on testosterone synthesis, operating at both the transcriptional level and the level of lipid metabolism, primarily through suppression of the AKT signaling pathway and its associated regulatory network (Xu et al. 2021, 2023; Zhu, Guo, et al. 2022). Genetic evidence further confirms that this process is specifically mediated by the melatonin receptors (melatonin receptor 1A) MEL1A and melatonin receptor 1B (MEL1B), as the knockdown of MEL1A and MEL1B reverses the inhibition of the cAMP/PKA pathway and testosterone synthesis by melatonin (Xu et al. 2021). However, this inhibitory regulation is potentially reversible under certain conditions. The most compelling evidence is derived from a glyphosate‐exposed rooster model, in which melatonin effectively alleviated suppression of testosterone synthesis by inhibiting mitophagy (Ren et al. 2024).

These seemingly contradictory findings precisely illustrate that the fundamental role of melatonin may not be as a simple stimulatory or inhibitory molecule but rather as a sophisticated modulator whose effects are highly dependent on physiological and pathological context. The ultimate output of its regulatory effects likely depends partially on the organism's intrinsic physiological/pathological state and inherent sensitivity to melatonin (Xu et al. 2021, 2023; Zhu, Guo, et al. 2022). This insight cautions against a direct extrapolation of findings from specific animal models—particularly species with reproductive strategies divergent from those of humans—to human applications without rigorous validation. Research using human induced pluripotent stem cells (hiPSCs) represents a promising approach for addressing challenges associated with different species in fundamental LOH studies. Studies confirm that hiPSCs can differentiate efficiently into functional Leydig‐like cells, and through the continuous optimization of experimental protocols, long‐lived, high‐testosterone‐producing Leydig‐like cells derived from hiPSCs can be successfully generated (Ishida et al. 2021; Sato et al. 2025).

Furthermore, the clinical value of melatonin may lie not in universally increasing testosterone levels but in restoring testosterone synthesis homeostasis under specific conditions (e.g., drug‐induced, environmental toxicant‐induced, or metabolic stress‐induced testicular dysfunction). These findings should help refocus future melatonin research from “whether it is effective” to “in which populations and under which pathological conditions it is effective,” thereby informing the development of precise clinical treatment strategies.

### 5.2 Exploration of Individualized Dosing Strategies

Within the current evidence framework, no prospective clinical trials have directly investigated melatonin treatment in patients with LOH using testosterone levels or improvements in related symptoms as primary endpoints, which substantially limits the exploration of its clinical application for LOH. Based on existing preclinical animal studies and limited clinical research, understanding the dose–response relationship, optimal timing of administration, and long‐term benefits of melatonin in ameliorating testicular or LC aging remains preliminary, with no established standardized dosing regimen. Furthermore, given the typical age of LOH onset, investigating whether dosing requirements exhibit age‐dependent variations and whether the choice of administration route differentially influences therapeutic efficacy is crucial.

Building on the established antioxidant and anti‐inflammatory properties of melatonin and the clinical evidence regarding its effects on symptoms associated with LOH, particular attention should be given to patient subgroups who may experience a greater therapeutic benefit. These subgroups likely include patients with marked OS, chronic inflammation, obesity, or characteristic symptoms such as sleep disturbances and ED. This approach is supported by human testicular single‐cell RNA sequencing data revealing more pronounced alterations in testicular aging in elderly individuals with a body mass index (BMI) exceeding 30 (Nie et al. 2022).

Moreover, considering that OS serves as a core driver of LC senescence, the therapeutic strategy of combining melatonin with antioxidants merits further exploration and investigation. Furthermore, screening for biomarkers related to OS and inflammation in patients with LOH will provide a basis for the precise application of melatonin therapy.

## 6 Conclusions and Future Perspectives

During aging, the senescence of LCs and the resulting decrease in testosterone synthesis constitute the core pathological basis of LOH. This degenerative process is driven not only by OS and mitochondrial dysfunction but also by epigenetic remodeling, the formation of the SASP, the deterioration of the stem cell niche, and circadian rhythm disruption. Together, these alterations represent a systemic, cascading progression from localized cellular damage to global testicular dysfunction.

Confronted with this complex pathological network, melatonin—an endogenous molecule with multi‐target anti‐aging potential—acts synergistically across multiple levels by precisely modulating redox homeostasis and mitochondrial function in LCs. Its mechanisms include upregulating key steroidogenic enzymes, regulating HPG axis function, and maintaining testicular microenvironmental homeostasis, thereby effectively delaying LC senescence and promoting the synthesis and secretion of endogenous testosterone.

Although the preclinical results are promising—particularly in large animal models in which a single administration improved testicular blood flow and increased testosterone levels (Samir et al. 2020; Shahat et al. 2022), providing novel insights for developing short‐term pulse therapies—significant challenges remain. A preliminary clinical trial showed that high‐dose melatonin (25 mg) administered at noon and bedtime significantly upregulated BMAL1 gene expression in Parkinson's disease patients, although its superiority over the placebo was limited and failed to improve clinical symptoms (Delgado‐Lara et al. 2020). These findings reveal the regulatory effect of melatonin on core clock genes, yet its specific mechanisms, its effect on testosterone synthesis, and its clinical benefits require further extensive investigation.

Key challenges in translating melatonin to an LOH treatment in clinical practice include the following: the relevance of animal models to human aging pathology, the establishment of optimal administration routes and dose–response relationships, the evaluation of long‐term safety, and the identification of precise clinical biomarkers for assessing efficacy. Addressing these bottlenecks will be crucial for future research.

In summary, melatonin represents not only a pharmaceutical option but also a paradigm shift from traditional “passive hormone supplementation” with TST toward “active cellular function restoration” targeting fundamental aging mechanisms. By addressing the underlying etiology of LC senescence in patients with LOH, melatonin has the potential to fundamentally modify the disease process. As the in‐depth understanding of its mechanisms improves and clinical translation pathways become clearer, melatonin is poised to lead to the development of new therapeutic strategies for LOH focused on cellular functional remodeling, resulting in sustained innovation and hope in this field.

## Author Contributions

All authors contributed to the literature review and writing of the manuscript. **Hui Wu:** writing – original draft, visualization, validation. **Gang Ning:** formal analysis, validation. **Mintao Jian:** validation. **Ajian Peng:** methodology. **Haoyu Wang:** data curation. **Bonan Li:** writing – review and editing, funding acquisition, visualization. **Xing Zhou:** writing – review and editing, funding acquisition, supervision. All the authors read and approved the final manuscript.

## Funding

This work was supported by National Natural Science Foundation of China, 82474525, 82505603, the Hunan Provincial Natural Outstanding Young People Science Foundation (Grant 2023JJ10032), the Natural Science Foundation of Changsha (Grant kq2502302) and the Traditional Chinese Medicine Research Program of Changsha (Grant SB2024‐115).

## Conflicts of Interest

The authors declare no conflicts of interest.

## Data Availability

The data that support the findings of this study are available on request from the corresponding author. The data are not publicly available due to privacy or ethical restrictions.

## References

[acel70492-bib-0001] Applewhite, J. , J. McCarter , G. Saffati , et al. 2025. “Testosterone Replacement Therapy in Men on Active Surveillance for Prostate Cancer.” Journal of Sexual Medicine 22, no. 3: 432–438. 10.1093/jsxmed/qdaf003. 39895152

[acel70492-bib-0002] Baburski, A. Z. , S. J. Sokanovic , M. M. Bjelic , S. M. Radovic , S. A. Andric , and T. S. Kostic . 2016. “Circadian Rhythm of the Leydig Cells Endocrine Function Is Attenuated During Aging.” Experimental Gerontology 73: 5–13. 10.1016/j.exger.2015.11.002. 26547053

[acel70492-bib-0003] Bahrami, N. , M. Goudarzi , A. Hosseinzadeh , S. Sabbagh , R. J. Reiter , and S. Mehrzadi . 2018. “Evaluating the Protective Effects of Melatonin on di(2‐Ethylhexyl) Phthalate‐Induced Testicular Injury in Adult Mice.” Biomedicine & Pharmacotherapy 108: 515–523. 10.1016/j.biopha.2018.09.044. 30243084

[acel70492-bib-0004] Bajgiran, F. A. , Z. K. Koohpar , and A. Salehzadeh . 2023. “Effects of N‐Acetylcysteine Supplementation on Oxidative Stress and Expression of Apoptosis‐Related Genes in Testicular Tissue of Rats Exposed to Lead.” Biological Trace Element Research 201, no. 5: 2407–2415. 10.1007/s12011-022-03325-0. 35761113

[acel70492-bib-0005] Bhasin, S. , and I. M. Thompson . 2024. “Prostate Risk and Monitoring During Testosterone Replacement Therapy.” Journal of Clinical Endocrinology and Metabolism 109, no. 8: 1975–1983. 10.1210/clinem/dgae334. 38753865

[acel70492-bib-0006] Bhasin, S. , T. G. Travison , K. M. Pencina , et al. 2023. “Prostate Safety Events During Testosterone Replacement Therapy in Men With Hypogonadism: A Randomized Clinical Trial.” JAMA Network Open 6, no. 12: e2348692. 10.1001/jamanetworkopen.2023.48692. 38150256 PMC10753401

[acel70492-bib-0007] Bozkurt, A. , M. Karabakan , B. K. Aktas , M. Gunay , E. Keskin , and E. Hirik . 2018. “Low Serum Melatonin Levels Are Associated With Erectile Dysfunction.” International Brazilian Journal Urology 44, no. 4: 794–799. 10.1590/S1677-5538.IBJU.2017.0663. PMC6092660 29757573

[acel70492-bib-0008] Cao, H. , Q. Xie , P. Luo , et al. 2024. “Di‐(2‐Ethylhexyl) Phthalate Exposure Induces Premature Testicular Senescence by Disrupting Mitochondrial Respiratory Chain Through STAT5B‐mitoSTAT3 in Leydig Cell.” Geroscience 46, no. 5: 4373–4396. 10.1007/s11357-024-01119-x. 38499958 PMC11336147

[acel70492-bib-0009] Chen, Y. , Y. Tang , B. Liu , et al. 2025. “Melatonin Alleviates Oxidative Stress and Inflammation of Leydig Cells of Min Pig Through SIRT1 Pathway.” Theriogenology 233: 112–122. 10.1016/j.theriogenology.2024.11.021. 39613495

[acel70492-bib-0010] Chen, Y. , J. Wang , D. Xu , et al. 2021. “m6A mRNA Methylation Regulates Testosterone Synthesis Through Modulating Autophagy in Leydig Cells.” Autophagy 17, no. 2: 457–475. 10.1080/15548627.2020.1720431. 31983283 PMC8007139

[acel70492-bib-0011] Chen, Z. , Z. Chen , J. Mo , Y. Chen , L. Chen , and C. Deng . 2025. “m6A RNA Methylation Modulates Autophagy by Targeting Map1lc3b in Bisphenol A Induced Leydig Cell Dysfunction.” Journal of Hazardous Materials 485: 136748. 10.1016/j.jhazmat.2024.136748. 39662354

[acel70492-bib-0012] Cheng, H. , X. Zhang , Y. Li , et al. 2024. “Age‐Related Testosterone Decline: Mechanisms and Intervention Strategies.” Reproductive Biology and Endocrinology 22, no. 1: 144. 10.1186/s12958-024-01316-5. 39543598 PMC11562514

[acel70492-bib-0013] Chhunchha, B. , E. Kubo , and D. P. Singh . 2020. “Clock Protein Bmal1 and Nrf2 Cooperatively Control Aging or Oxidative Response and Redox Homeostasis by Regulating Rhythmic Expression of Prdx6.” Cells 9, no. 8: 1861. 10.3390/cells9081861. 32784474 PMC7463585

[acel70492-bib-0014] Chi, A. , B. Yang , H. Dai , et al. 2024. “Stem Leydig Cells Support Macrophage Immunological Homeostasis Through Mitochondrial Transfer in Mice.” Nature Communications 15, no. 1: 2120. 10.1038/s41467-024-46190-2. PMC10924100 38459012

[acel70492-bib-0015] Cho, Y. , and Y. K. Kim . 2024. “ROS‐Mediated Cytoplasmic Localization of CARM1 Induces Mitochondrial Fission Through DRP1 Methylation.” Redox Biology 73: 103212. 10.1016/j.redox.2024.103212. 38838552 PMC11179627

[acel70492-bib-0016] Chung, J. , H. Chen , and B. Zirkin . 2021. “Sirt1 and Nrf2: Regulation of Leydig Cell Oxidant/Antioxidant Intracellular Environment and Steroid Formation.” Biology of Reproduction 105, no. 5: 1307–1316. 10.1093/biolre/ioab150. 34363387 PMC8598996

[acel70492-bib-0017] Chung, J. Y. , H. Chen , A. Midzak , A. L. Burnett , V. Papadopoulos , and B. R. Zirkin . 2013. “Drug Ligand‐Induced Activation of Translocator Protein (TSPO) Stimulates Steroid Production by Aged Brown Norway Rat Leydig Cells.” Endocrinology 154, no. 6: 2156–2165. 10.1210/en.2012-2226. 23525219 PMC3740486

[acel70492-bib-0018] Corona, G. , W. Vena , A. Pizzocaro , et al. 2022. “Testosterone Supplementation and Bone Parameters: A Systematic Review and Meta‐Analysis Study.” Journal of Endocrinological Investigation 45, no. 5: 911–926. 10.1007/s40618-021-01702-5. 35041193

[acel70492-bib-0019] Cruciani, S. , G. Garroni , R. Pala , et al. 2022. “Melatonin Finely Tunes Proliferation and Senescence in Hematopoietic Stem Cells.” European Journal of Cell Biology 101, no. 3: 151251. 10.1016/j.ejcb.2022.151251. 35772322

[acel70492-bib-0020] Cruickshank, M. , J. Hudson , R. Hernández , et al. 2024. “The Effects and Safety of Testosterone Replacement Therapy for Men With Hypogonadism: The TestES Evidence Synthesis and Economic Evaluation.” Health Technology Assessment 28, no. 43: 1–210. 10.3310/JRYT3981. PMC11404359 39248210

[acel70492-bib-0021] Cui, L. , X. Nie , Y. Guo , et al. 2025. “Single‐Cell Transcriptomic Atlas of the Human Testis Across the Reproductive Lifespan.” Nature Aging 5, no. 4: 658–674. 10.1038/s43587-025-00824-2. 40033047 PMC12003174

[acel70492-bib-0022] Dalian, G. , L. Zhihui , K. Rongda , et al. 2026. “Deficiency of NR2C2 Accelerates Senescence of Testicular Leydig Cells and Infertility in Male Mice.” Cellular Signalling 138: 112284. 10.1016/j.cellsig.2025.112284. 41317934

[acel70492-bib-0023] de Almeida, L. G. N. , H. Thode , Y. Eslambolchi , et al. 2022. “Matrix Metalloproteinases: From Molecular Mechanisms to Physiology, Pathophysiology, and Pharmacology.” Pharmacological Reviews 74, no. 3: 712–768. 10.1124/pharmrev.121.000349. 35738680

[acel70492-bib-0024] de Araújo Silva, E. F. , J. A. d. S. Gomes , M. L. F. de Oliveira , et al. 2023. “Protective Effect of Exogenous Melatonin on Testicular Histopathology and Histomorphometry of Adult Rats With Domperidone‐Induced Hyperprolactinemia.” Reproductive Biology 23, no. 3: 100791. 10.1016/j.repbio.2023.100791. 37517145

[acel70492-bib-0025] Dehdari Ebrahimi, N. , S. Shojaei‐Zarghani , E. Taherifard , et al. 2023. “Protective Effects of Melatonin Against Physical Injuries to Testicular Tissue: A Systematic Review and Meta‐Analysis of Animal Models.” Frontiers in Endocrinology 14: 1123999. 10.3389/fendo.2023.1123999. 36798664 PMC9927015

[acel70492-bib-0026] Delgado‐Lara, D. L. , G. V. González‐Enríquez , B. M. Torres‐Mendoza , et al. 2020. “Effect of Melatonin Administration on the PER1 and BMAL1 Clock Genes in Patients With Parkinson's Disease.” Biomedicine & Pharmacotherapy 129: 110485. 10.1016/j.biopha.2020.110485. 32768967

[acel70492-bib-0027] Deng, S. L. , Z. P. Wang , C. Jin , et al. 2018. “Melatonin Promotes Sheep Leydig Cell Testosterone Secretion in a Co‐Culture With Sertoli Cells.” Theriogenology 106: 170–177. 10.1016/j.theriogenology.2017.10.025. 29073541

[acel70492-bib-0028] Dong, Y. , J. Zhao , Q. Zhu , H. Liu , J. Wang , and W. Lu . 2020. “Melatonin Inhibits the Apoptosis of Rooster Leydig Cells by Suppressing Oxidative Stress via AKT‐Nrf2 Pathway Activation.” Free Radical Biology & Medicine 160: 1–12. 10.1016/j.freeradbiomed.2020.06.024. 32758663

[acel70492-bib-0029] Du, Z. , S. Xu , S. Hu , et al. 2018. “Melatonin Attenuates Detrimental Effects of Diabetes on the Niche of Mouse Spermatogonial Stem Cells by Maintaining Leydig Cells.” Cell Death & Disease 9, no. 10: 968. 10.1038/s41419-018-0956-4. 30237484 PMC6148071

[acel70492-bib-0030] Englund, D. A. , A. Jolliffe , Z. Aversa , et al. 2023. “p21 Induces a Senescence Program and Skeletal Muscle Dysfunction.” Molecular Metabolism 67: 101652. 10.1016/j.molmet.2022.101652. 36509362 PMC9800630

[acel70492-bib-0031] Fan, R. , X. Peng , L. Xie , et al. 2022. “Importance of Bmal1 in Alzheimer's Disease and Associated Aging‐Related Diseases: Mechanisms and Interventions.” Aging Cell 21, no. 10: e13704. 10.1111/acel.13704. 36056774 PMC9577946

[acel70492-bib-0032] Fotovat, A. , B. Samadzadeh , M. Ayati , et al. 2022. “The Effect of Melatonin on Improving the Benign Prostatic Hyperplasia Urinary Symptoms, a Randomized Clinical Trial.” Urology Journal 19, no. 5: 406–411. 10.22037/uj.v18i.6761. 34746997

[acel70492-bib-0033] Franci, L. , A. Tubita , F. M. Bertolino , et al. 2022. “MAPK15 Protects From Oxidative Stress‐Dependent Cellular Senescence by Inducing the Mitophagic Process.” Aging Cell 21, no. 7: e13620. 10.1111/acel.13620. 35642724 PMC9282834

[acel70492-bib-0034] Franci, L. , G. Vallini , F. M. Bertolino , et al. 2024. “MAPK15 Controls Cellular Responses to Oxidative Stress by Regulating NRF2 Activity and Expression of Its Downstream Target Genes.” Redox Biology 72: 103131. 10.1016/j.redox.2024.103131. 38555711 PMC10998232

[acel70492-bib-0035] Galansky, L. B. , J. A. Levy , and A. L. Burnett . 2022. “Testosterone and Male Sexual Function.” Urologic Clinics of North America 49, no. 4: 627–635. 10.1016/j.ucl.2022.07.006. 36309419

[acel70492-bib-0036] Gao, Y. , X. Wu , S. Zhao , et al. 2019. “Melatonin Receptor Depletion Suppressed hCG‐Induced Testosterone Expression in Mouse Leydig Cells.” Cellular & Molecular Biology Letters 24: 21. 10.1186/s11658-019-0147-z. 30915128 PMC6416941

[acel70492-bib-0037] Garza, S. , L. Chen , M. Galano , et al. 2022. “Mitochondrial Dynamics, Leydig Cell Function, and Age‐Related Testosterone Deficiency.” FASEB Journal 36, no. 12: e22637. 10.1096/fj.202201026R. 36349989

[acel70492-bib-0038] Grillo, G. , S. Falvo , D. Latino , et al. 2024. “Polystyrene Microplastics Impair the Functions of Cultured Mouse Leydig (TM3) and Sertoli (TM4) Cells by Inducing Mitochondrial‐Endoplasmic Reticulum Damage.” Ecotoxicology and Environmental Safety 274: 116202. 10.1016/j.ecoenv.2024.116202. 38479314

[acel70492-bib-0039] Gul, Y. S. , O. Kose , A. Altin , et al. 2024. “Melatonin Supports Nonsurgical Periodontal Treatment in Patients With Type 2 Diabetes Mellitus and Periodontitis: A Randomized Clinical Trial.” Journal of Periodontology 95, no. 9: 832–841. 10.1002/JPER.23-0335. 38055628

[acel70492-bib-0040] Guo, S. , L. Fu , C. Yin , et al. 2024. “ROS‐Induced Gingival Fibroblast Senescence: Implications in Exacerbating Inflammatory Responses in Periodontal Disease.” Inflammation 47, no. 6: 1918–1935. 10.1007/s10753-024-02014-5. 38630168

[acel70492-bib-0041] Hainan, C. , C. Jinsi , H. Li , et al. 2025. “Mitochondria‐Localized Nestin Protects Mesenchymal Stem Cells From Senescence by Maintaining Cristae Structure and Function.” Advanced Science (Weinheim, Germany) 12, no. 46: e07759. 10.1002/advs.202507759. PMC12697773 40990041

[acel70492-bib-0042] Han, D. , J. Yao , W. Chen , et al. 2023. “Altered Transcriptomic and Metabolomic Profiles of Testicular Interstitial Fluid During Aging in Mice.” Theriogenology 200: 86–95. 10.1016/j.theriogenology.2023.02.004. 36773384

[acel70492-bib-0043] He, J. , J. Li , Y. Li , et al. 2024. “Single‐Cell Transcriptomics Identifies Senescence‐Associated Secretory Phenotype (SASP) Features of Testicular Aging in Human.” Aging (Albany NY) 16, no. 4: 3350–3362. 10.18632/aging.205538. 38349859 PMC10929807

[acel70492-bib-0044] Hossein, A. , G. Moreno Denisse , I. Bloom Samuel , N. Lucas , A. Lesniewski Lisa , and J. Donato Anthony . 2025. “MitoQ Reduces Senescence Burden in Doxorubicin‐Treated Endothelial Cells by Reducing Mitochondrial ROS and DNA Damage.” American Journal of Physiology. Heart and Circulatory Physiology 329, no. 5: H1154–H1161. 10.1152/ajpheart.00568.2025. 41026856 PMC12758503

[acel70492-bib-0045] Hou, X. , J. Shi , L. Sun , et al. 2022. “The Involvement of ERK1/2 and p38 MAPK in the Premature Senescence of Melanocytes Induced by H_2_O_2_ Through a p53‐Independent p21 Pathway.” Journal of Dermatological Science 105, no. 2: 88–97. 10.1016/j.jdermsci.2022.01.002. 35042627

[acel70492-bib-0046] Hu, C. , Y. Feng , G. Huang , et al. 2024. “Melatonin Prevents EAAC1 Deletion‐Induced Retinal Ganglion Cell Degeneration by Inhibiting Apoptosis and Senescence.” Journal of Pineal Research 76, no. 1: e12916. 10.1111/jpi.12916. 37786968

[acel70492-bib-0047] Huang, D. , W. Wei , F. Xie , X. Zhu , L. Zheng , and Z. Lv . 2018. “Steroidogenesis Decline Accompanied With Reduced Antioxidation and Endoplasmic Reticulum Stress in Mice Testes During Ageing.” Andrologia 50, no. 1: e12816. 10.1111/and.12816. 28488286

[acel70492-bib-0048] Huang, J. , L. Sun , Y. Yin , et al. 2025. “High Matrix Stiffness Triggers Testosterone Decline in Aging Males by Disrupting Stem Leydig Cell Pool Homeostasis.” Cell Reports 44, no. 9: 116207. 10.1016/j.celrep.2025.116207. 40884795

[acel70492-bib-0049] Huang, W. , J. Zhang , C. Miao , et al. 2024. “Aflatoxin B1‐Induced Testosterone Biosynthesis Disorder via the ROS/AMPK Signaling Pathway in Male Mice.” Journal of Agricultural and Food Chemistry 72, no. 11: 5955–5965. 10.1021/acs.jafc.3c08769. 38451160

[acel70492-bib-0050] Ishida, T. , M. Koyanagi‐Aoi , D. Yamamiya , et al. 2021. “Differentiation of Human Induced Pluripotent Stem Cells Into Testosterone‐Producing Leydig‐Like Cells.” Endocrinology 162, no. 12: bqab202. 10.1210/endocr/bqab202. 34549267

[acel70492-bib-0051] Isidori, A. M. , A. Aversa , A. Calogero , et al. 2022. “Adult‐ and Late‐Onset Male Hypogonadism: The Clinical Practice Guidelines of the Italian Society of Andrology and Sexual Medicine (SIAMS) and the Italian Society of Endocrinology (SIE).” Journal of Endocrinological Investigation 45, no. 12: 2385–2403. 10.1007/s40618-022-01859-7. 36018454 PMC9415259

[acel70492-bib-0052] Jallouli, S. , S. Ghroubi , N. Bouattour , et al. 2025. “Effects of Melatonin Supplementation on Muscle Strength, Manual Dexterity, and Postural Balance in Patients Living With Multiple Sclerosis–A Randomized Controlled Trial.” Journal of Dietary Supplements 22, no. 2: 236–261. 10.1080/19390211.2024.2449030. 39849681

[acel70492-bib-0053] Jiang, X. , B. Liu , Z. Nie , et al. 2021. “The Role of m6A Modification in the Biological Functions and Diseases.” Signal Transduction and Targeted Therapy 6, no. 1: 74. 10.1038/s41392-020-00450-x. 33611339 PMC7897327

[acel70492-bib-0054] Kang, H. , Z. Hong , M. Zhong , et al. 2019. “Piezo1 Mediates Angiogenesis Through Activation of MT1‐MMP Signaling.” American Journal of Physiology. Cell Physiology 316, no. 1: C92–C103. 10.1152/ajpcell.00346.2018. 30427721 PMC6383143

[acel70492-bib-0055] Kruse, R. , S. J. Petersson , L. L. Christensen , et al. 2020. “Effect of Long‐Term Testosterone Therapy on Molecular Regulators of Skeletal Muscle Mass and Fibre‐Type Distribution in Aging Men With Subnormal Testosterone.” Metabolism 112: 154347. 10.1016/j.metabol.2020.154347. 32853647

[acel70492-bib-0056] Kumar, J. , R. Verma , and C. Haldar . 2021. “Melatonin Ameliorates Bisphenol S Induced Testicular Damages by Modulating Nrf‐2/HO‐1 and SIRT‐1/FOXO‐1 Expressions.” Environmental Toxicology 36, no. 3: 396–407. 10.1002/tox.23045. 33098627

[acel70492-bib-0057] Kwon, H. Y. , H. Lee , J. S. Choi , et al. 2025. “Fermented *Morinda citrifolia* Extract Improves Late‐Onset Hypogonadism in Aged Rats.” Biomedicine & Pharmacotherapy 189: 118256. 10.1016/j.biopha.2025.118256. 40516329

[acel70492-bib-0058] Lei, T. , Y. Yang , and W. Yang . 2025. “Luteinizing Hormone Regulates Testosterone Production, Leydig Cell Proliferation, Differentiation, and Circadian Rhythm During Spermatogenesis.” International Journal of Molecular Sciences 26, no. 8: 3548. 10.3390/ijms26083548. 40332028 PMC12027374

[acel70492-bib-0059] Li, J. , X. Dong , L. Gao , et al. 2024. “Mitochondrial Malfunction‐Initiated Leydig Cell Premature Senescence Partially Participates in 1‐Nitropyrene‐Evoked Downregulation of Steroidogenic Synthases in Testes.” Free Radical Biology and Medicine 225: 456–468. 10.1016/j.freeradbiomed.2024.10.291. 39426755

[acel70492-bib-0060] Li, R. , Y. Li , H. Zuo , G. Pei , S. Huang , and Y. Hou . 2024. “Alzheimer's Amyloid‐β Accelerates Cell Senescence and Suppresses SIRT1 in Human Neural Stem Cells.” Biomolecules 14, no. 2: 189. 10.3390/biom14020189. 38397428 PMC10886734

[acel70492-bib-0061] Li, Y. , W. Liang , Y. Han , W. Zhao , S. Wang , and C. Qin . 2022. “Triterpenoids and Polysaccharides From Ganoderma Lucidum Improve the Histomorphology and Function of Testes in Middle‐Aged Male Mice by Alleviating Oxidative Stress and Cellular Apoptosis.” Nutrients 14, no. 22: 4733. 10.3390/nu14224733. 36432421 PMC9696538

[acel70492-bib-0062] Li, Y. , C. Zhang , H. Cheng , et al. 2024. “FOXO4‐DRI Improves Spermatogenesis in Aged Mice Through Reducing Senescence‐Associated Secretory Phenotype Secretion From Leydig Cells.” Experimental Gerontology 195: 112522. 10.1016/j.exger.2024.112522. 39025385

[acel70492-bib-0063] Li, Z. , L. Hao , S. Chen , et al. 2024. “Forkhead Box C1 Promotes the Pathology of Osteoarthritis in Subchondral Bone Osteoblasts via the Piezo1/YAP Axis.” Cellular Signalling 124: 111463. 10.1016/j.cellsig.2024.111463. 39396563

[acel70492-bib-0064] Liang, Y. , Y. Yang , C. Lu , et al. 2024. “Polystyrene Nanoplastics Exposure Triggers Spermatogenic Cell Senescence via the Sirt1/ROS Axis.” Ecotoxicology and Environmental Safety 279: 116461. 10.1016/j.ecoenv.2024.116461. 38763051

[acel70492-bib-0065] Lincoff, A. M. , S. Bhasin , P. Flevaris , et al. 2023. “Cardiovascular Safety of Testosterone‐Replacement Therapy.” New England Journal of Medicine 389, no. 2: 107–117. 10.1056/NEJMoa2215025. 37326322

[acel70492-bib-0066] Liu, F. , Z. Chen , J. Zhao , Y. Zhang , J. Ma , and W. Li . 2022. “T2DM‐Elicited Oxidative Stress Represses MTA3 Expression in Mouse Leydig Cells.” Reproduction 163, no. 5: 267–280. 10.1530/REP-21-0413. 35239504

[acel70492-bib-0067] Liu, H. , Z. Guo , Z. Zang , et al. 2025. “Saffron Extract Alleviates D‐Gal‐Induced Late‐Onset Hypogonadism by Activating the PI3K‐Akt‐Nrf2 Signaling Pathway.” Journal of Ethnopharmacology 340: 119273. 10.1016/j.jep.2024.119273. 39710157

[acel70492-bib-0068] Liu, J. , Q. Guan , S. Li , Q. Qi , and X. Pan . 2025. “Melatonin Alleviates MBP‐Induced Oxidative Stress and Apoptosis in TM3 Cells via the SIRT1/PGC‐1α Signaling Pathway.” International Journal of Molecular Sciences 26, no. 12: 5910. 10.3390/ijms26125910. 40565373 PMC12193403

[acel70492-bib-0069] Liu, Y. J. , X. B. Shen , N. Yu , et al. 2021. “Prevalence of Late‐Onset Hypogonadism Among Middle‐Aged and Elderly Males in China: Results From a National Survey.” Asian Journal of Andrology 23, no. 2: 170–177. 10.4103/aja.aja_59_20. 33154202 PMC7991815

[acel70492-bib-0070] Lu, L. , J. Liu , J. Wang , C. Lian , Z. Wang , and L. Wang . 2022. “Glyphosate‐Induced Mitochondrial Reactive Oxygen Species Overproduction Activates Parkin‐Dependent Mitophagy to Inhibit Testosterone Synthesis in Mouse Leydig Cells.” Environmental Pollution 314: 120314. 10.1016/j.envpol.2022.120314. 36183875

[acel70492-bib-0071] Lu, X. , J. Liu , J. Li , Q. Yang , and J. Zhang . 2018. “Melatonin Therapy Adds Extra Benefit to Varicecelectomy in Terms of Sperm Parameters, Hormonal Profile and Total Antioxidant Capacity: A Placebo‐Controlled, Double‐Blind Trial.” Andrologia 50, no. 6: e13033. 10.1111/and.13033. 29740842

[acel70492-bib-0072] Luo, D. , X. Qi , X. Xu , L. Yang , C. Yu , and Q. Guan . 2023. “Involvement of p38 MAPK in Leydig Cell Aging and Age‐Related Decline in Testosterone.” Frontiers in Endocrinology 14: 1088249. 10.3389/fendo.2023.1088249. 36950685 PMC10025507

[acel70492-bib-0073] Lv, Z. , C. Liu , P. Wang , and Y. Chen . 2024. “Dysregulation of Mitochondrial Dynamics and Mitophagy Are Involved in High‐Fat Diet‐Induced Steroidogenesis Inhibition.” Journal of Lipid Research 65, no. 10: 100639. 10.1016/j.jlr.2024.100639. 39236859 PMC11467671

[acel70492-bib-0074] Ma, J. , H. Yang , L. Liu , Y. Wan , and F. Wang . 2021. “Melatonin Alleviated Oxidative Stress Induced by Energy Restriction on Sheep Leydig Cells Through Sirt1/Sod2 Pathway.” Theriogenology 173: 83–92. 10.1016/j.theriogenology.2021.07.011. 34352672

[acel70492-bib-0075] Meng, J. , L. Xu , B. Ma , et al. 2025. “GABARAPL1 Is Essential for ACR‐Induced Autophagic Cell Death of Mouse Leydig Cells.” Ecotoxicology and Environmental Safety 289: 117426. 10.1016/j.ecoenv.2024.117426. 39626489

[acel70492-bib-0076] Mohammadi, N. , M. Alizadeh , S. Akbarzadeh , et al. 2025. “Melatonin Administered Postoperatively Lowers Oxidative Stress and Inflammation and Significantly Recovers Heart Function in Patients Undergoing CABG Surgery.” European Journal of Medical Research 30, no. 1: 585. 10.1186/s40001-025-02789-9. 40619439 PMC12232798

[acel70492-bib-0077] Monageng, E. , U. Offor , N. B. Takalani , K. Mohlala , and C. S. Opuwari . 2023. “A Review on the Impact of Oxidative Stress and Medicinal Plants on Leydig Cells.” Antioxidants (Basel) 12, no. 8: 1559. 10.3390/antiox12081559. 37627554 PMC10451682

[acel70492-bib-0078] Morozumi, W. , K. Aoshima , S. Inagaki , et al. 2022. “Piezo1 Activation Induces Fibronectin Reduction and PGF2α Secretion via Arachidonic Acid Cascade.” Experimental Eye Research 215: 108917. 10.1016/j.exer.2021.108917. 34973946

[acel70492-bib-0079] Morvaridzadeh, M. , E. Sadeghi , S. Agah , et al. 2020. “Effect of Melatonin Supplementation on Oxidative Stress Parameters: A Systematic Review and Meta‐Analysis.” Pharmacological Research 161: 105210. 10.1016/j.phrs.2020.105210. 33007423

[acel70492-bib-0080] Mularoni, V. , V. Esposito , S. Di Persio , et al. 2020. “Age‐Related Changes in Human Leydig Cell Status.” Human Reproduction 35, no. 12: 2663–2676. 10.1093/humrep/deaa271. 33094328

[acel70492-bib-0081] Naelitz, B. D. , L. Momtazi‐Mar , S. Vallabhaneni , et al. 2025. “Testosterone Replacement Therapy and Spermatogenesis in Reproductive Age Men.” Nature Reviews. Urology 22, no. 10: 703–719. 10.1038/s41585-025-01032-8. 40346275

[acel70492-bib-0082] Neves, B. V. D. , F. Lorenzini , D. Veronez , E. P. de Miranda , G. D. Neves , and R. de Fraga . 2017. “Numeric and Volumetric Changes in Leydig Cells During Aging of Rats.” Acta Cirúrgica Brasileira 32, no. 10: 807–815. 10.1590/s0102-865020170100000002. 29160367

[acel70492-bib-0083] Nguyen, B. T. , C. Lin , T. Chang , et al. 2023. “Melatonin Inhibits Chondrosarcoma Cell Proliferation and Metastasis by Enhancing miR‐520f‐3p Production and Suppressing MMP7 Expression.” Journal of Pineal Research 75, no. 1: e12872. 10.1111/jpi.12872. 37057370

[acel70492-bib-0084] Nie, X. , S. K. Munyoki , M. Sukhwani , et al. 2022. “Single‐Cell Analysis of Human Testis Aging and Correlation With Elevated Body Mass Index.” Developmental Cell 57, no. 9: 1160–1176. 10.1016/j.devcel.2022.04.004. 35504286 PMC9090997

[acel70492-bib-0085] Ning, G. , B. Li , H. Wu , et al. 2025. “Regulation of Testosterone Synthesis by Circadian Clock Genes and Its Research Progress in Male Diseases.” Asian Journal of Andrology 27, no. 5: 564–573. 10.4103/aja20258. 40101130 PMC12422579

[acel70492-bib-0086] Niu, L. , P. Yang , B. Zhu , et al. 2024. “Inhibition of the RIP3/MLKL/TRPM7 Necroptotic Pathway Ameliorates Diabetes Mellitus‐Induced Erectile Dysfunction by Reducing Cell Death, Fibrosis, and Inflammation.” Frontiers in Pharmacology 15: 1436013. 10.3389/fphar.2024.1436013. 39329120 PMC11424535

[acel70492-bib-0087] Park, C. , H. J. Cha , M. Y. Kim , et al. 2022. “Phloroglucinol Attenuates DNA Damage and Apoptosis Induced by Oxidative Stress in Human Retinal Pigment Epithelium ARPE‐19 Cells by Blocking the Production of Mitochondrial ROS.” Antioxidants 11, no. 12: 2353. 10.3390/antiox11122353. 36552561 PMC9774705

[acel70492-bib-0088] Pavlovic, M. V. , D. Z. Marinkovic , S. A. Andric , and T. S. Kostic . 2022. “The Cost of the Circadian Desynchrony on the Leydig Cell Function.” Scientific Reports 12, no. 1: 15520. 10.1038/s41598-022-19889-9. 36109553 PMC9478133

[acel70492-bib-0089] Pencina, K. M. , T. G. Travison , G. R. Cunningham , et al. 2024. “Effect of Testosterone Replacement Therapy on Sexual Function and Hypogonadal Symptoms in Men With Hypogonadism.” Journal of Clinical Endocrinology and Metabolism 109, no. 2: 569–580. 10.1210/clinem/dgad484. 37589949

[acel70492-bib-0090] Prasad, K. , S. C. Kaul , R. Wadhwa , K. P. Guruprasad , and K. Satyamoorthy . 2025. “Cellular Oxidative Stress and Sirtuins Mediate Regulation of Senescence and Neuronal Differentiation by Withaferin A.” Free Radical Biology & Medicine 233: 174–185. 10.1016/j.freeradbiomed.2025.03.038. 40154756

[acel70492-bib-0091] Qi, Q. , L. Feng , J. Liu , D. Xu , G. Wang , and X. Pan . 2024. “Melatonin Alleviates BPA‐Induced Testicular Apoptosis and Endoplasmic Reticulum Stress.” Frontiers in Bioscience (Landmark Edition) 29, no. 3: 95. 10.31083/j.fbl2903095. 38538260

[acel70492-bib-0092] Qi, Q. , J. Yang , S. Li , et al. 2024. “Melatonin Alleviates Oxidative Stress Damage in Mouse Testes Induced by Bisphenol A.” Frontiers in Cell and Developmental Biology 12: 1338828. 10.3389/fcell.2024.1338828. 38440074 PMC10910031

[acel70492-bib-0093] Qi, Z. , W. Yang , B. Xue , et al. 2024. “ROS‐Mediated Lysosomal Membrane Permeabilization and Autophagy Inhibition Regulate Bleomycin‐Induced Cellular Senescence.” Autophagy 20, no. 9: 2000–2016. 10.1080/15548627.2024.2353548. 38762757 PMC11346523

[acel70492-bib-0094] Qin, D. , H. Cai , C. He , et al. 2021. “Melatonin Relieves Heat‐Induced Spermatocyte Apoptosis in Mouse Testes by Inhibition of ATF6 and PERK Signaling Pathways.” Zoological Research 42, no. 4: 514–524. 10.24272/j.issn.2095-8137.2021.041. 34254745 PMC8317181

[acel70492-bib-0095] Qin, Z. , J. Song , J. Huang , et al. 2023. “Mitigation of Triptolide‐Induced Testicular Sertoli Cell Damage by Melatonin via Regulating the Crosstalk Between SIRT1 and NRF2.” Phytomedicine 118: 154945. 10.1016/j.phymed.2023.154945. 37437414

[acel70492-bib-0096] Rasmussen, R. S. , M. Midttun , B. Zerahn , et al. 2024. “Testosterone and Resistance Training Improved Physical Performance and Reduced Fatigue in Frail Older Men: 1 Year Follow‐Up of a Randomized Clinical Trial.” Aging Male 27, no. 1: 2403519. 10.1080/13685538.2024.2403519. 39289825

[acel70492-bib-0097] Ren, Y. , Q. Liang , C. Lian , W. Zhang , and L. Wang . 2024. “Melatonin Alleviates Glyphosate‐Induced Testosterone Synthesis Inhibition via Targeting Mitochondrial Function in Roosters.” Environmental Pollution 348: 123828. 10.1016/j.envpol.2024.123828. 38522604

[acel70492-bib-0098] Riviere, E. , S. P. Rossi , Y. E. Tavalieri , et al. 2020. “Melatonin Daily Oral Supplementation Attenuates Inflammation and Oxidative Stress in Testes of Men With Altered Spermatogenesis of Unknown Aetiology.” Molecular and Cellular Endocrinology 515: 110889. 10.1016/j.mce.2020.110889. 32622722

[acel70492-bib-0099] Rossi, S. P. , M. E. Matzkin , E. Riviere , et al. 2023. “Melatonin Improves Oxidative State and Lactate Metabolism in Rodent Sertoli Cells.” Molecular and Cellular Endocrinology 576: 112034. 10.1016/j.mce.2023.112034. 37516434

[acel70492-bib-0100] Sahu, A. , and R. Verma . 2023. “Bisphenol S Dysregulates Thyroid Hormone Homeostasis; Testicular Survival, Redox and Metabolic Status: Ameliorative Actions of Melatonin.” Environmental Toxicology and Pharmacology 104: 104300. 10.1016/j.etap.2023.104300. 37866414

[acel70492-bib-0101] Saidi, A. O. , C. O. Akintayo , C. L. Atuma , et al. 2022. “Melatonin Supplementation Preserves Testicular Function by Attenuating Lactate Production and Oxidative Stress in High Fat Diet‐Induced Obese Rat Model.” Theriogenology 187: 19–26. 10.1016/j.theriogenology.2022.02.029. 35500423

[acel70492-bib-0102] Salonia, A. , P. Capogrosso , L. Boeri , et al. 2025. “European Association of Urology Guidelines on Male Sexual and Reproductive Health: 2025 Update on Male Hypogonadism, Erectile Dysfunction, Premature Ejaculation, and Peyronie's Disease.” European Urology 88, no. 1: 76–102. 10.1016/j.eururo.2025.04.010. 40340108

[acel70492-bib-0103] Samir, H. , A. S. Mandour , F. Radwan , et al. 2023. “Effect of Acute Melatonin Injection on Metabolomic and Testicular Artery Hemodynamic Changes and Circulating Hormones in Shiba Goats Under Sub‐Tropical Environmental Conditions.” Animals 13, no. 11: 1794. 10.3390/ani13111794. 37889744 PMC10252098

[acel70492-bib-0104] Samir, H. , P. Nyametease , M. Elbadawy , K. Nagaoka , K. Sasaki , and G. Watanabe . 2020. “Administration of Melatonin Improves Testicular Blood Flow, Circulating Hormones, and Semen Quality in Shiba Goats.” Theriogenology 146: 111–119. 10.1016/j.theriogenology.2020.01.053. 32078960

[acel70492-bib-0105] Sang, J. , Z. Ji , H. Li , et al. 2024. “Triclosan Inhibits Testosterone Biosynthesis in Adult Rats via Inducing m6A Methylation‐Mediated Autophagy.” Environment International 190: 108827. 10.1016/j.envint.2024.108827. 38908274

[acel70492-bib-0106] Sato, K. , M. Koyanagi‐Aoi , K. Uehara , et al. 2025. “Efficient Differentiation of Human iPSCs Into Leydig‐Like Cells Capable of Long‐Term Stable Secretion of Testosterone.” Stem Cell Reports 20, no. 2: 102392. 10.1016/j.stemcr.2024.102392. 39824187 PMC11864132

[acel70492-bib-0107] Sawada, N. , M. Nomiya , M. Zarifpour , T. Mitsui , M. Takeda , and K. Andersson . 2016. “Melatonin Improves Erectile Function in Rats With Chronic Lower Body Ischemia.” Journal of Sexual Medicine 13, no. 2: 179–186. 10.1016/j.jsxm.2015.12.018. 26803454

[acel70492-bib-0108] Shahat, A. M. , J. C. Thundathil , and J. P. Kastelic . 2022. “Melatonin Improves Testicular Hemodynamics and Sperm Quality in Rams Subjected to Mild Testicular Heat Stress.” Theriogenology 188: 163–169. 10.1016/j.theriogenology.2022.05.029. 35691188

[acel70492-bib-0109] Shao, J. , J. Wang , X. Wen , et al. 2023. “Effects of Aging and Macrophages on Mice Stem Leydig Cell Proliferation and Differentiation In Vitro.” Frontiers Endocrinology 14: 1139281. 10.3389/fendo.2023.1139281. PMC10083278 37051204

[acel70492-bib-0110] Siraj, Y. , D. Aprile , N. Alessio , G. Peluso , G. D. Bernardo , and U. Galderisi . 2024. “IGFBP7 Is a Key Component of the Senescence‐Associated Secretory Phenotype (SASP) That Induces Senescence in Healthy Cells by Modulating the Insulin, IGF, and Activin A Pathways.” Cell Communication and Signaling 22, no. 1: 540. 10.1186/s12964-024-01921-2. 39533382 PMC11558980

[acel70492-bib-0111] Snyder, P. J. 2022. “Symptoms of Late‐Onset Hypogonadism in Men.” Endocrinology and Metabolism Clinics of North America 51, no. 4: 755–760. 10.1016/j.ecl.2022.04.001. 36244691

[acel70492-bib-0112] Sokanovic, S. J. , A. Z. Baburski , Z. Kojic , M. L. J. Medar , S. A. Andric , and T. S. Kostic . 2021. “Aging‐Related Increase of cGMP Disrupts Mitochondrial Homeostasis in Leydig Cells.” Journals of Gerontology. Series A, Biological Sciences and Medical Sciences 76, no. 2: 177–186. 10.1093/gerona/glaa132. 32459846

[acel70492-bib-0113] Sugumaran, R. , K. S. S. Krishna , J. Saibaba , S. K. Narayan , S. Sandhiya , and M. Rajeswari . 2024. “Melatonin on Sleep in Parkinson's Disease: A Randomized Double Blind Placebo Controlled Trial.” Sleep Medicine 124: 502–509. 10.1016/j.sleep.2024.10.020. 39437460

[acel70492-bib-0114] Tang, X. , Y. Xu , N. Ou , Y. Tang , and H. Chen . 2025. “Update on Late‐Onset Hypogonadism: Current Concepts, Controversies, Clinical Diagnosis, Pathogenesis, and Treatment Approaches.” Sex Med Rev 13, no. 4: 652–662. 10.1093/sxmrev/qeaf043. 40751587

[acel70492-bib-0115] Tang, Z. , J. Song , Z. Yu , et al. 2019. “Melatonin Treatment Ameliorates Hyperhomocysteinemia‐Induced Impairment of Erectile Function in a Rat Model.” Journal of Sexual Medicine 16, no. 10: 1506–1517. 10.1016/j.jsxm.2019.07.003. 31439521

[acel70492-bib-0116] Travicic, D. Z. , D. Miljkovic , S. A. Andric , and T. S. Kostic . 2025. “Circadian Disruption Impairs Leydig Cell Maturation and Reproductive Development in Male Rats.” Reproductive Biology and Endocrinology 23, no. 1: 104. 10.1186/s12958-025-01440-w. 40676622 PMC12273390

[acel70492-bib-0117] Umaimah, Z. , S. Lai Agnes , P. Jaclyn , et al. 2025. “Melatonin Supplementation and Oxidative DNA Damage Repair Capacity Among Night Shift Workers: A Randomised Placebo‐Controlled Trial.” Occupational and Environmental Medicine 82, no. 1: 1–6. 10.1136/oemed-2024-109824. 39993913

[acel70492-bib-0118] Venugopal, S. P. 2019. “Effect of Melatonin on the Onset of Puberty in Male Juvenile Rats.” Anat Cell Biol 52, no. 3: 286–295. 10.5115/acb.18.122. 31598358 PMC6773899

[acel70492-bib-0119] Wang, B. , J. Han , J. H. Elisseeff , and M. Demaria . 2024. “The Senescence‐Associated Secretory Phenotype and Its Physiological and Pathological Implications.” Nature Reviews. Molecular Cell Biology 25, no. 12: 958–978. 10.1038/s41580-024-00727-x. 38654098

[acel70492-bib-0120] Wang, C. , K. Yang , F. Gao , M. Zheng , and X. Fu . 2025. “FTO Regulates Testosterone Secretion in Leydig Cells: Insights Into the Role of m6A Modifications and the Therapeutic Potential of hCG.” Reproductive Biology and Endocrinology 23, no. 1: 121. 10.1186/s12958-025-01456-2. 40993667 PMC12462280

[acel70492-bib-0121] Wang, L. , Z. Lu , J. Zhao , et al. 2021. “Selective Oxidative Stress Induces Dual Damage to Telomeres and Mitochondria in Human T Cells.” Aging Cell 20, no. 12: e13513. 10.1111/acel.13513. 34752684 PMC8672791

[acel70492-bib-0122] Wang, M. , J. Xu , Z. Zhao , et al. 2023. “Triphenyl Phosphate Induced Apoptosis of Mice Testicular Leydig Cells and TM3 Cells Through ROS‐Mediated Mitochondrial Fusion Inhibition.” Ecotoxicology and Environmental Safety 256: 114876. 10.1016/j.ecoenv.2023.114876. 37027944

[acel70492-bib-0123] Wang, P. , S. Zhang , S. Lin , and Z. Lv . 2022. “Melatonin Ameliorates Diabetic Hyperglycaemia‐Induced Impairment of Leydig Cell Steroidogenic Function Through Activation of SIRT1 Pathway.” Reproductive Biology and Endocrinology 20, no. 1: 117. 10.1186/s12958-022-00991-6. 35962432 PMC9373359

[acel70492-bib-0124] Wang, S. , H. Lu , M. Su , et al. 2024. “Bisphenol H Exposure Disrupts Leydig Cell Function in Adult Rats via Oxidative Stress‐Mediated m6A Modifications: Implications for Reproductive Toxicity.” Ecotoxicology and Environmental Safety 285: 117061. 10.1016/j.ecoenv.2024.117061. 39303633

[acel70492-bib-0125] Wen, F. , J. Gao , G. Zhang , et al. 2025. “ROS‐DRP1‐Mediated Excessive Mitochondrial Fission and Autophagic Flux Inhibition Contribute to Heat Stress‐Induced Apoptosis in Goat Sertoli Cells.” Journal of Animal Science and Biotechnology 16, no. 1: 58. 10.1186/s40104-025-01180-2. 40234984 PMC12001645

[acel70492-bib-0126] Wolosowicz, M. , S. Prokopiuk , and T. W. Kaminski . 2024. “The Complex Role of Matrix Metalloproteinase‐2 (MMP‐2) in Health and Disease.” International Journal of Molecular Sciences 25, no. 24: 13691. 10.3390/ijms252413691. 39769454 PMC11728377

[acel70492-bib-0127] Wu, D. , G. Zhu , K. Zhao , et al. 2024. “Age‐Related Decline in Melatonin Contributes to Enhanced Osteoclastogenesis via Disruption of Redox Homeostasis.” Molecular Medicine (Cambridge, Mass) 30, no. 1: 10. 10.1186/s10020-024-00779-x. 38216878 PMC10785421

[acel70492-bib-0128] Xia, K. , P. Luo , J. Yu , et al. 2024. “Single‐Cell RNA Sequencing Reveals Transcriptomic Landscape and Potential Targets for Human Testicular Ageing.” Human Reproduction 39, no. 10: 2189–2209. 10.1093/humrep/deae199. 39241251 PMC11447013

[acel70492-bib-0129] Xu, G. , Z. Yuan , J. Hou , et al. 2021. “Prolonging Photoperiod Promotes Testosterone Synthesis of Leydig Cells by Directly Targeting Local Melatonin System in Rooster Testes.” Biology of Reproduction 105, no. 5: 1317–1329. 10.1093/biolre/ioab155. 34401899

[acel70492-bib-0130] Xu, G. , J. Zhao , H. Liu , J. Wang , and W. Lu . 2019. “Melatonin Inhibits Apoptosis and Oxidative Stress of Mouse Leydig Cells via a SIRT1‐Dependent Mechanism.” Molecules 24, no. 17: 3084. 10.3390/molecules24173084. 31450679 PMC6749417

[acel70492-bib-0131] Xu, H. , J. Pu , Y. Teng , et al. 2023. “Melatonin Inhibits Testosterone Synthesis in Rooster Leydig Cells by Targeting CXCL14 Through miR‐7481‐3p.” International Journal of Molecular Sciences 24, no. 23: 16552. 10.3390/ijms242316552. 38068875 PMC10706588

[acel70492-bib-0132] Xu, H. , J. Zhao , W. Lyu , and J. Wang . 2025. “A Review on the Epigenetic Regulation of Testosterone Synthesis in Leydig Cells.” Molecular and Cellular Biochemistry 480: 6127–6133. 10.1007/s11010-025-05366-0. 40767883

[acel70492-bib-0133] Yang, D. , Y. Wei , Q. Lu , et al. 2021. “Melatonin Alleviates LPS‐Induced Endoplasmic Reticulum Stress and Inflammation in Spermatogonial Stem Cells.” Journal of Cellular Physiology 236, no. 5: 3536–3551. 10.1002/jcp.30088. 32996162

[acel70492-bib-0134] Yang, L. , J. Cheng , D. Xu , et al. 2023. “Melatonin Ameliorates Diquat‐Induced Testicular Toxicity via Reducing Oxidative Stress, Inhibiting Apoptosis, and Maintaining the Integrity of Blood‐Testis Barrier in Mice.” Toxics 11, no. 2: 160. 10.3390/toxics11020160. 36851035 PMC9958747

[acel70492-bib-0135] Yang, L. , S. Liu , P. Song , et al. 2025. “DEHP‐Mediated Oxidative Stress Leads to Impaired Testosterone Synthesis in Leydig Cells Through the cAMP/PKA/SF‐1/StAR Pathway.” Environmental Pollution 366: 125503. 10.1016/j.envpol.2024.125503. 39657860

[acel70492-bib-0136] Yang, L. , T. Ma , L. Zhao , et al. 2021. “Circadian Regulation of Apolipoprotein Gene Expression Affects Testosterone Production in Mouse Testis.” Theriogenology 174: 9–19. 10.1016/j.theriogenology.2021.06.023. 34416563

[acel70492-bib-0137] Yang, M. , S. Guan , J. Tao , et al. 2021. “Melatonin Promotes Male Reproductive Performance and Increases Testosterone Synthesis in Mammalian Leydig Cells†.” Biology of Reproduction 104, no. 6: 1322–1336. 10.1093/biolre/ioab046. 33709108

[acel70492-bib-0138] Yang, S. , M. Chen , J. Meng , et al. 2024. “Melatonin Alleviates di‐Butyl Phthalate (DBP)‐induced Ferroptosis of Mouse Leydig Cells via Inhibiting Sp2/VDAC2 Signals.” Environmental Research 247: 118221. 10.1016/j.envres.2024.118221. 38246300

[acel70492-bib-0139] Yang, S. , Y. Yang , L. Xu , C. Hao , and J. Chen . 2025. “DAPK3 Is Essential for DBP‐Induced Autophagy of Mouse Leydig Cells.” Adv Sci (Weinh) 12, no. 17: e2413936. 10.1002/advs.202413936. 40047320 PMC12061289

[acel70492-bib-0140] Yao, S. , X. Wei , W. Deng , et al. 2022. “Nestin‐Dependent Mitochondria‐ER Contacts Define Stem Leydig Cell Differentiation to Attenuate Male Reproductive Ageing.” Nature Communications 13, no. 1: 4020. 10.1038/s41467-022-31755-w. PMC9276759 35821241

[acel70492-bib-0141] Yu, J. , V. Jaiswal , Y. Jang , M. Park , and H. J. Lee . 2025. “ *Salvia miltiorrhiza* Activates Nrf2/HO‐1 Signaling and Restores Steroidogenesis in Leydig TM3 Cells and an Aging Rat Model.” Biomedicine & Pharmacotherapy 189: 118297. 10.1016/j.biopha.2025.118297. 40582100

[acel70492-bib-0142] Yu, S. , K. Cui , P. Wu , et al. 2022. “Melatonin Prevents Experimental Central Serous Chorioretinopathy in Rats.” Journal of Pineal Research 73, no. 1: e12802. 10.1111/jpi.12802. 35436360

[acel70492-bib-0143] Yu, S. , X. Wang , P. Geng , et al. 2017. “Melatonin Regulates PARP1 to Control the Senescence‐Associated Secretory Phenotype (SASP) in Human Fetal Lung Fibroblast Cells.” Journal of Pineal Research 63, no. 1: e12405. 10.1111/jpi.12405. 28247536

[acel70492-bib-0144] Yuanyuan, L. , C. Jingjing , C. Yaru , et al. 2026. “Pyraclostrobin Induces Brain Oxidative Stress, Apoptosis and Inflammation Through Mitochondrial Phosphorylation‐Induced ROS Activation of the p38 MAPK Pathway.” Pesticide Biochemistry and Physiology 216, no. 1: 106787. 10.1016/j.pestbp.2025.106787. 41326079

[acel70492-bib-0145] Zhang, B. , Y. Zhong , J. Du , et al. 2024. “1,2‐Dichloroethane Induces Testicular Pyroptosis by Activating piR‐Mmu‐1019957/IRF7 Pathway and the Protective Effects of Melatonin.” Environment International 184: 108480. 10.1016/j.envint.2024.108480. 38341879

[acel70492-bib-0146] Zhang, H. , K. Li , Y. Wang , et al. 2025. “ROS Regulates Circadian Rhythms by Modulating Ezh2 Interactions With Clock Proteins.” Redox Biology 81: 103526. 10.1016/j.redox.2025.103526. 39952198 PMC11875201

[acel70492-bib-0147] Zhang, J. , Y. Fang , D. Tang , et al. 2022. “Activation of MT1/MT2 to Protect Testes and Leydig Cells Against Cisplatin‐Induced Oxidative Stress Through the SIRT1/Nrf2 Signaling Pathway.” Cells 11, no. 10: 1690. 10.3390/cells11101690. 35626727 PMC9139217

[acel70492-bib-0148] Zhang, L. , F. Ding , X. Wu , et al. 2023. “Melatonin Ameliorates Glyphosate‐ and Hard Water‐Induced Renal Tubular Epithelial Cell Senescence via PINK1‐Parkin‐Dependent Mitophagy.” Ecotoxicology and Environmental Safety 255: 114719. 10.1016/j.ecoenv.2023.114719. 37032573

[acel70492-bib-0149] Zhang, M. , B. Ma , S. Yang , J. Wang , and J. Chen . 2023. “Bisphenol A (BPA) Induces Apoptosis of Mouse Leydig Cells via Oxidative Stress.” Environmental Toxicology 38, no. 2: 312–321. 10.1002/tox.23690. 36315628

[acel70492-bib-0150] Zhang, M. , J. Wang , C. Deng , et al. 2017. “Transplanted Human p75‐Positive Stem Leydig Cells Replace Disrupted Leydig Cells for Testosterone Production.” Cell Death & Disease 8, no. 10: e3123. 10.1038/cddis.2017.531. 29022899 PMC5680910

[acel70492-bib-0151] Zhang, X. , X. Tang , T. Gao , et al. 2025. “Melatonin Receptor 1a Alleviates Sleep Fragmentation‐Aggravated Testicular Injury in T2DM by Suppression of TAB1/TAK1 Complex Through FGFR1.” Acta Pharmaceutica Sinica B 15, no. 7: 3591–3610. 10.1016/j.apsb.2025.05.018. 40698135 PMC12278403

[acel70492-bib-0152] Zhang, Y. , W. Huang , Z. Zheng , et al. 2021. “Cigarette Smoke‐Inactivated SIRT1 Promotes Autophagy‐Dependent Senescence of Alveolar Epithelial Type 2 Cells to Induce Pulmonary Fibrosis.” Free Radical Biology & Medicine 166: 116–127. 10.1016/j.freeradbiomed.2021.02.013. 33609723

[acel70492-bib-0153] Zhao, M. , X. Song , H. Chen , et al. 2022. “Melatonin Prevents Chondrocyte Matrix Degradation in Rats With Experimentally Induced Osteoarthritis by Inhibiting Nuclear Factor‐κB via SIRT1.” Nutrients 14, no. 19: 3966. 10.3390/nu14193966. 36235621 PMC9571821

[acel70492-bib-0154] Zhao, T. , J. Wang , Y. Wu , et al. 2021. “Increased m6A Modification of RNA Methylation Related to the Inhibition of Demethylase FTO Contributes to MEHP‐Induced Leydig Cell Injury.” Environmental Pollution 268: 115627. 10.1016/j.envpol.2020.115627. 33010548

[acel70492-bib-0155] Zhao, Y. , X. Li , H. Zhang , et al. 2022. “Phthalate‐Induced Testosterone/Androgen Receptor Pathway Disorder on Spermatogenesis and Antagonism of Lycopene.” Journal of Hazardous Materials 439: 129689. 10.1016/j.jhazmat.2022.129689. 36104915

[acel70492-bib-0156] Zhao, Z. , S. Mei , Q. Zheng , et al. 2023. “Melatonin or Vitamin C Attenuates Lead Acetate‐Induced Testicular Oxidative and Inflammatory Damage in Mice by Inhibiting Oxidative Stress Mediated NF‐κB Signaling.” Ecotoxicology and Environmental Safety 264: 115481. 10.1016/j.ecoenv.2023.115481. 37716076

[acel70492-bib-0157] Zheng, X. , X. Zhang , L. Tan , et al. 2024. “Sirt1 m6A Modification‐Evoked Leydig Cell Senescence Promotes cd‐Induced Testosterone Decline.” Ecotoxicology and Environmental Safety 284: 116884. 10.1016/j.ecoenv.2024.116884. 39153281

[acel70492-bib-0158] Zhou, S. M. , J. Z. Li , H. Q. Chen , et al. 2023. “FTO‐Nrf2 Axis Regulates Bisphenol F‐Induced Leydig Cell Toxicity in an m6A‐YTHDF2‐Dependent Manner.” Environmental Pollution 325: 121393. 10.1016/j.envpol.2023.121393. 36878272

[acel70492-bib-0159] Zhu, Q. , L. Guo , W. An , et al. 2022. “Melatonin Inhibits Testosterone Synthesis in Roosters Leydig Cells by Regulating Lipolysis of Lipid Droplets.” Theriogenology 189: 118–126. 10.1016/j.theriogenology.2022.06.016. 35753225

[acel70492-bib-0160] Zhu, R. , X. Ji , X. Wu , et al. 2022. “Melatonin Antagonizes Ovarian Aging via YTHDF2‐MAPK‐NF‐κB Pathway.” Genes Dis 9, no. 2: 494–509. 10.1016/j.gendis.2020.08.005. 35224163 PMC8843885

[acel70492-bib-0161] Ziegler, D. V. , N. Martin , and D. Bernard . 2021. “Cellular Senescence Links Mitochondria‐ER Contacts and Aging.” Communications Biology 4, no. 1: 1323. 10.1038/s42003-021-02840-5. 34819602 PMC8613202

